# Hepatic TRPC3 loss contributes to chronic alcohol consumption-induced hepatic steatosis and liver injury in mice

**DOI:** 10.1093/lifemeta/load050

**Published:** 2023-12-18

**Authors:** Qinchao Ding, Rui Guo, Liuyi Hao, Qing Song, Ai Fu, Shanglei Lai, Tiantian Xu, Hui Zhuge, Kaixin Chang, Yanli Chen, Haibin Wei, Daxi Ren, Zhaoli Sun, Zhenyuan Song, Xiaobing Dou, Songtao Li

**Affiliations:** School of Public Health, Zhejiang Chinese Medical University, Hangzhou, Zhejiang 310053, China; College of Animal Science, Zhejiang University, Hangzhou, Zhejiang 310058, China; School of Public Health, Zhejiang Chinese Medical University, Hangzhou, Zhejiang 310053, China; School of Public Health, Zhejiang Chinese Medical University, Hangzhou, Zhejiang 310053, China; School of Public Health, Zhejiang Chinese Medical University, Hangzhou, Zhejiang 310053, China; Department of Kinesiology and Nutrition, University of Illinois at Chicago, Chicago, IL 60612, United States; School of Life Science, Zhejiang Chinese Medical University, Hangzhou, Zhejiang 310053, China; School of Life Science, Zhejiang Chinese Medical University, Hangzhou, Zhejiang 310053, China; School of Public Health, Zhejiang Chinese Medical University, Hangzhou, Zhejiang 310053, China; School of Life Science, Zhejiang Chinese Medical University, Hangzhou, Zhejiang 310053, China; School of Life Science, Zhejiang Chinese Medical University, Hangzhou, Zhejiang 310053, China; School of Life Science, Zhejiang Chinese Medical University, Hangzhou, Zhejiang 310053, China; School of Life Science, Zhejiang Chinese Medical University, Hangzhou, Zhejiang 310053, China; College of Animal Science, Zhejiang University, Hangzhou, Zhejiang 310058, China; Department of Surgery, Johns Hopkins University School of Medicine, Baltimore, MD 21205, United States; Department of Kinesiology and Nutrition, University of Illinois at Chicago, Chicago, IL 60612, United States; School of Life Science, Zhejiang Chinese Medical University, Hangzhou, Zhejiang 310053, China; School of Public Health, Zhejiang Chinese Medical University, Hangzhou, Zhejiang 310053, China

**Keywords:** TRPC3, alcohol-associated liver disease, miR-339-5p, hepatic steatosis, liver injury, CAMKK2/AMPK

## Abstract

Emerging evidence discloses the involvement of calcium channel protein in the pathological process of liver diseases. Transient receptor potential cation channel subfamily C member 3 (TRPC3), a ubiquitously expressed non-selective cation channel protein, controls proliferation, inflammation, and immune response via operating calcium influx in various organs. However, our understanding on the biofunction of hepatic TRPC3 is still limited. The present study aims to clarify the role and potential mechanism(s) of TRPC3 in alcohol-associated liver disease (ALD). We recently found that TRPC3 expression plays an important role in the disease process of ALD. Alcohol exposure led to a significant reduction of hepatic TRPC3 in patients with alcohol-related hepatitis (AH) and ALD models. Antioxidants (N-acetylcysteine and mitoquinone) intervention improved alcohol-induced suppression of TRPC3 via a miR-339-5p-involved mechanism. TRPC3 loss robustly aggravated the alcohol-induced hepatic steatosis and liver injury in mouse liver; this was associated with the suppression of Ca^2+^/calmodulin-dependent protein kinase kinase 2 (CAMKK2)/AMP-activated protein kinase (AMPK) and dysregulation of genes related to lipid metabolism. TRPC3 loss also enhanced hepatic inflammation and early fibrosis-like change in mice. Replenishing hepatic TRPC3 effectively reversed chronic alcohol-induced detrimental alterations in ALD mice. Briefly, chronic alcohol exposure-induced TRPC3 reduction contributes to the pathological development of ALD via suppression of the CAMKK2/AMPK pathway. Oxidative stress-stimulated miR-339-5p upregulation contributes to alcohol-reduced TRPC3. TRPC3 is the requisite and a potential target to defend alcohol consumption-caused ALD.

## Introduction

Harmful alcohol consumption leads to about 5.9% of all deaths (3.3 million deaths) worldwide annually and is positively associated with multiple disorders and diseases, among which, alcohol-associated liver disease (ALD) has become one of the most serious health issues globally, accounting for 47.9% of all liver cirrhosis deaths and 30% of all hepatocellular carcinoma (HCC) deaths [1]. The spectrum of ALD ranges from steatosis (fatty liver), steatohepatitis (ASH), characterized by hepatocyte cell death and immune cell activation and infiltration, to fibrosis/cirrhosis, even HCC [2].

It has been well established that excessive reactive oxygen species (ROS), generated from either alcohol catabolism, which is primarily regulated by alcohol dehydrogenases and microsomal oxidases in hepatocytes, or NADPH oxidase activation in Kupffer cells, are mechanistically involved in the pathogenesis of ALD [3, 4]. In the early stage of ALD, oxidative stress promotes hepatic fat accumulation (steatosis) through (i) promoting hepatic lipid synthesis via activating sterol regulatory element-binding protein 1c (SREBP-1c), 1-aminocyclopropane-1-carboxylate (ACC), fatty acid synthase (FAS), and acyl-CoA:diacylglycerol acyltransferase (DGAT); (ii) retarding lipid catabolism via suppressing peroxisome proliferator-activated receptor α (PPARα), AMP-activated protein kinase (AMPK), and carnitine palmitoyltransferase I (CPT1); (iii) enhancing extrahepatic lipid uptake by the liver via upregulating low-density lipoprotein (VLDL) receptor (VLDLR), fatty acid transport protein (FATP), and cluster of differentiation 36 (CD36); and (iv) lowering the secretion of lipoproteins (VLDL) [5]. Simple hepatic steatosis is the initial and relatively reversible stage of ALD. Although significant progress has been made in understanding the pathological basis of this stage, there is still a lack of effective therapeutic approaches in the clinic currently. Therefore, clarifying potential mechanism(s) underscoring the initiation and progression of the disease remains an urgent task for ALD improvement.

Impaired Ca^2+^ influx and Ca^2+^-mediated signal transduction in ALD have been documented both experimentally and clinically [6, 7], implying the significance of Ca^2+^ channels in the pathological progress of ALD. Transient receptor potential cation channel subfamily C (TRPC) is an evolutionarily conserved non-selective cation channel protein and is mainly located in the cytomembrane with six transmembrane-spanning segments [8]. Four subfamilies (total of seven members) of TRPC have been identified, including TRPC1, TRPC2, TRPC4/5, and TRPC3/6/7 according to sequence homology [8]. TRPC3 is one of the most well-studied TRPC members and is ubiquitously expressed in both excitable and non-excitable cells, including hepatocytes. Recent evidence showed that TRPC3 was required to maintain survival, prevent stimuli-induced apoptosis, and promote immune responses via controlling Ca^2+^ influx [9–11]. However, it has also been reported that TRPC3 upregulation contributed to pro-inflammatory response and aggravated the development of cardiovascular disease and tumors [12–14]. It is apparent that the function of TRPC3 is stimulus- and cell-type-specific, with both promoting and preventive activities being reported, depending on the type of diseases being investigated [15]. Nevertheless, limited studies have been conducted to examine the role of hepatic TRPC3 expression and function in ALD.

In this study, we report for the first time that TRPC3 is significantly decreased in live samples from both alcohol-related hepatitis (AH) patients and ALD mice. Liver-specific TRPC3 silencing aggravates alcohol-induced hepatic steatosis and liver injury by suppressing Ca^2+^/calmodulin-dependent protein kinase kinase 2 (CAMKK2)/AMPK signaling pathway. Notably, liver-specific TRPC3 overexpression prevents ALD development in mice. Our further mechanistic analysis reveals that oxidative stress-upregulated micro (miR)-339-5p contributes to alcohol-reduced TRPC3. Altogether, our data provide evidence supporting the potential translational application of TRPC3 as a therapeutic target for ALD.

## Results

### Alcohol consumption reduces hepatic TRPC3 levels in both mouse and human livers

**Figure 1:**
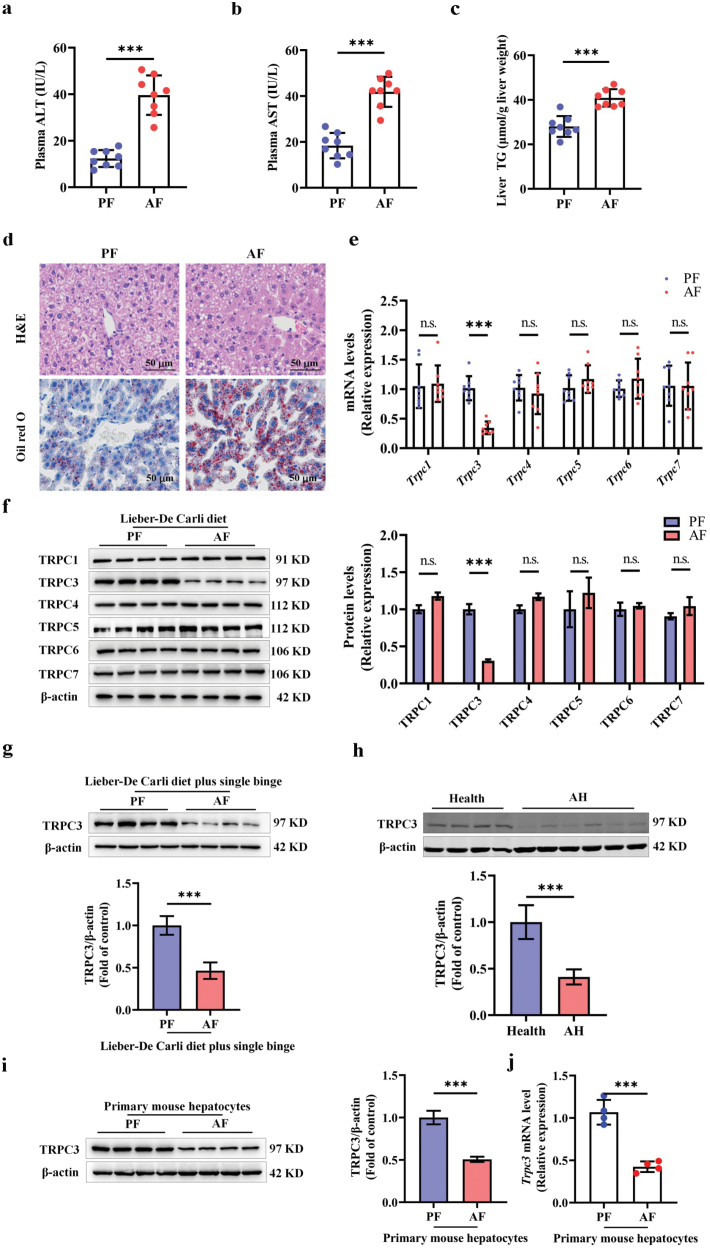
Alcohol consumption reduces hepatic TRPC3 expression. (a and b) Plasma ALT and AST levels. (c) Liver TG contents. (d) Liver H&E and Oil red O staining. (e and f) mRNA and protein expressions of TRPCs in various ALD models. (g) TRPC3 protein expression in Lieber-De Carli diet plus single binge model. (H) TRPC3 protein expression in patients with AH (*n* = 6) and healthy individuals (*n* = 4). (i and j) Primary hepatocytes were isolated from the livers of Lieber-De Carli diet-fed PF and AF mice. TRPC3 expression was detected. Protein band intensity was quantified by ImageJ. Data are presented as means ± SD (*n* = 4–8 for animals). ^***^P < 0.001 compared with corresponding control. n.s. represents no statistical difference.

Limited investigations have been performed to test the effect of chronic alcohol intake on liver TRPC channels. Here, a widely used ALD model established by feeding mice with a Lieber-De Carli ethanol-containing liquid diet was employed to examine the effect of alcohol consumption on hepatic TRPC expression (Fig. 1a–d). As shown in Fig. 1e and f, chronic alcohol consumption significantly decreased hepatic TRPC3 expression without affecting other TRPC members. The alcohol-induced TRPC3 downregulation was also observed in the Lieber-De Carli diet plus single binge model (Fig. 1g). To validate the clinical relevance of our observations in mice, TRPC3 protein abundance in liver samples obtained from AH patients and healthy individuals was subsequently examined. As shown in Fig. 1h, hepatic TRPC3 protein expression was markedly decreased in patients with AH when compared with that in healthy individuals. Further, TRPC3 expression was reduced in primary hepatocytes isolated from the livers of chronic alcohol-fed (AF, fed with the Lieber-De Carli alcohol liquid diet) mice (Fig. 1i and j). In addition, ethanol-induced reduction of TRPC3 expression was observed in both mouse AML-12 hepatocytes and human VL-17A hepatocytes (Supplementary Fig. S1). Considering the sequence homology for TRPC3, TRPC6, and TRPC7, the specificity for the TRPC3 antibody was confirmed using its special siRNA (Supplementary Fig. S2).

### Oxidative stress-stimulated miR-339-5p upregulation contributes to alcohol-induced TRPC3 downregulation

**Figure 2:**
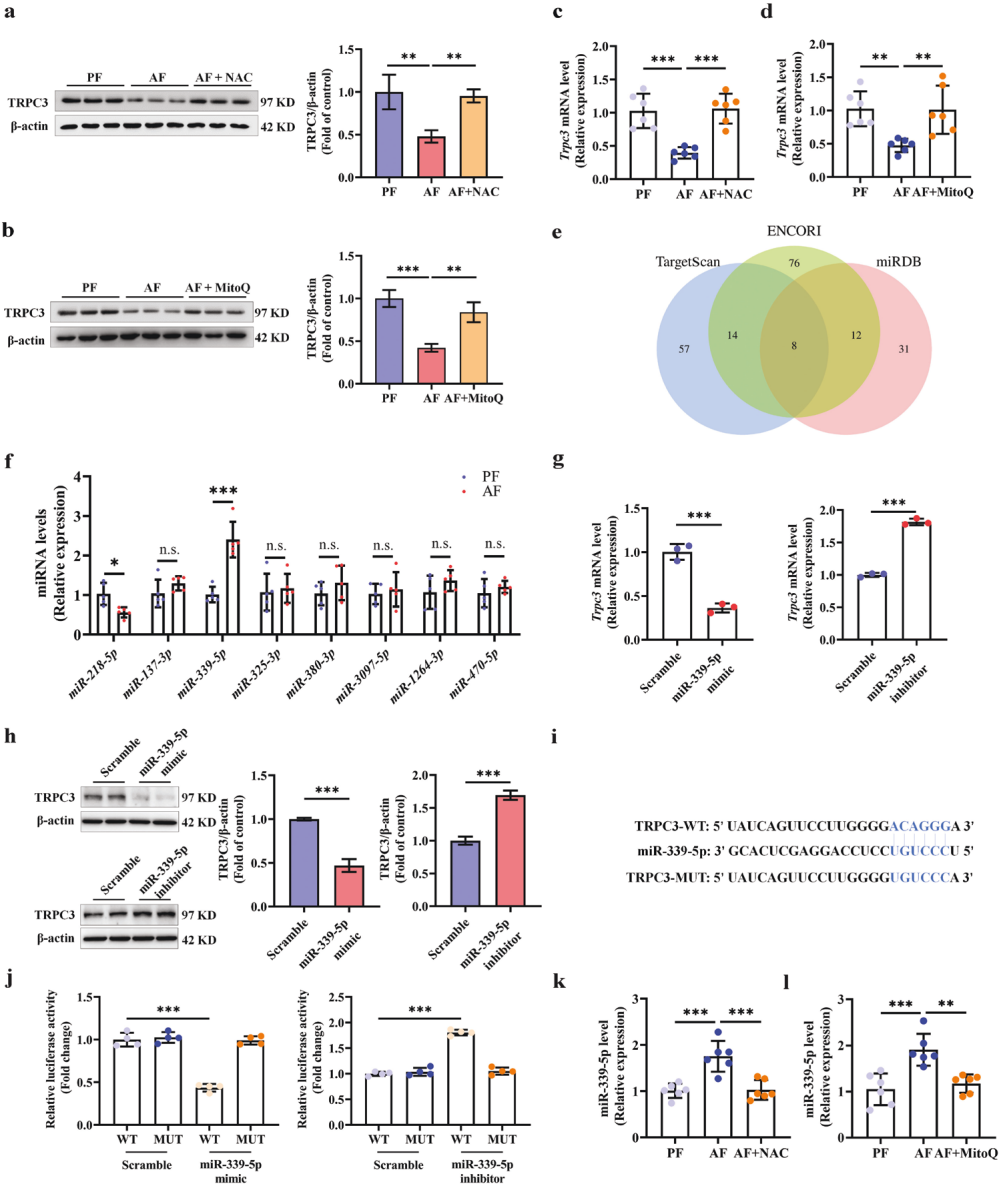
Oxidative stress-stimulated miR-339-5p upregulation contributes to alcohol-reduced TRPC3. (a–d) TRPC3 protein and mRNA expressions in chronic AF mice supplemented with either NAC (40 mg/kg body weight/day) or MitoQ (5 mg/kg body weight/day), respectively. (e) Venn plot for the eight overlapped miRNAs. (f) Relative expressions of miRNAs in chronic AF mouse liver. (g and h) AML-12 hepatocytes were transfected with miR-339-5p mimic or inhibitor for 48 h. TRPC3 mRNA and protein expressions were detected. (i) Alignments of miR-339-5p binding to the 3ʹ-UTRs of *Trpc3* mRNA. (j) HEK293T cells were co-transfected with miR-339-5p mimic or inhibitor and TRPC3-3ʹ-UTR reporter. Relative luciferase activity was assayed in HEK293T cells. (k and l) The relative expression of miR-339-5p was detected in chronic AF mouse liver supplemented with either NAC or MitoQ, respectively. Protein band intensity was quantified by ImageJ. Data are presented as means ± SD (*n* = 6–8 for animals; *n* = 3–4 for cultured cells). ^**^P < 0.01, ^***^P < 0.001 compared with corresponding control. n.s. represents no statistical difference.

To explore the potential mechanism(s) underlying alcohol-induced TRPC3 downregulation in hepatocytes and the liver, several pathways, including oxidative stress, endoplasmic reticulum (ER) stress, mitogen-activated protein kinases (MAPKs), hypoxia-inducible factor-1α (Hif-1α), nuclear factor erythroid 2-related factor 2 (Nrf2), etc., which have been documented to implicate in alcohol-induced hepatic steatosis [16–19], were screened using multiple specific chemical inhibitors. We identified that in hepatocytes, N-acetylcysteine (NAC), a glutathione precursor and potent antioxidant, abolished alcohol-induced TRPC3 reduction (Supplementary Fig. S3). Moreover, in mice, administration of both NAC and mitoquinone (MitoQ) rescued alcohol-induced hepatic TRPC3 loss (Fig. 2a–d). miRNAs have been reported to be implicated in ALD pathogenesis [20]. Here, we predicted miRNA candidates targeting *Trpc3* by filtering three databases, including ENCORI (via ENCORI website), TargetScan via TargetScanHuman 8.0 website, and miRDB via miRDB online database. After taking the intersections, eight miRNA candidates were selected for further testing in ALD mouse liver (Fig. 2e). Our data showed that miR-339-5p was significantly upregulated in the liver of AF mice when compared to that in pair-fed (PF, fed with isocaloric maltose dextrin control liquid diet) group (Fig. 2f). miR-339-5p overexpression significantly downregulated TRPC3 expression at both mRNA and protein levels in AML-12 hepatocytes, while miR-339-5p knockdown led to a robust enhancement of TRPC3 expression (Fig. 2g and h). Furthermore, sequence matching analysis revealed a strong affinity between the seed sequence of miR-339-5p and the 3ʹ-untranslated region (UTR) of *Trpc3* (Fig. 2i). To provide direct evidence supporting our hypothesis, The luciferase reporting system was utilized to investigate the effects of miR-339-5p mimic and inhibitor transfection on luciferase expression from the luciferase-TRPC3-3ʹ-UTR construct in HEK293T cells. We observed that miR-339-5p mimic transfection decreased luciferase expression, while miR-339-5p inhibitor transfection enhanced it (Fig. 2j). Notably, these effects were not observed when mutant structures were employed (Fig. 2j). Additionally, either NAC or MitoQ intervention reversed the alcohol-induced increase of miR-339-5p in ALD mouse liver, respectively (Fig. 2k and l). These data collaboratively implied that oxidative stress-stimulated miR-339-5p upregulation was involved in alcohol-caused TRPC3 downregulation.

### TRPC3 knockdown deteriorates alcohol-induced hepatic injury

**Figure 3:**
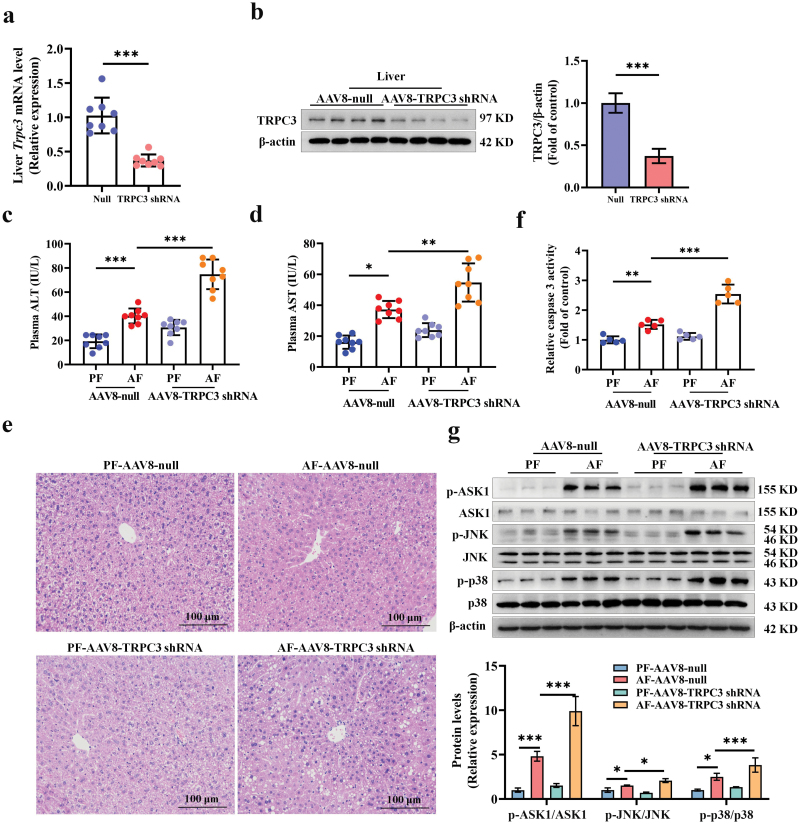
Liver-specific TRPC3 knockdown deteriorates alcohol-induced hepatic injury in mice. Hepatocyte-specific TRPC3 knockdown mice were generated by caudal vein injection with recombinant AAV8 gene transfer vectors bearing a liver-specific promoter (TBG) in combination with mouse TRPC3 shRNA sequence. Mice injected with null-vector were served as control. (a and b) Hepatic TRPC3 knockdown efficiency in mRNA and protein levels was detected. (c and d) Plasma ALT and AST levels. (e) Liver H&E staining. (f) Caspase 3 activity in mouse liver. (g) Phosphorylated-ASK1, -JNK, and -p38 protein expressions were detected in mouse livers. Protein band intensity was quantified by ImageJ. Data are presented as means ± SD (*n* = 6–8 for animals). ^*^P < 0.05, ^**^P < 0.01, ^***^P < 0.001 compared with corresponding control.

To determine the pathogenic contribution of hepatic TRPC3 loss in ALD development in response to chronic alcohol exposure, liver-specific TRPC3 knockdown was achieved in mice via caudal vein delivery of TRPC3 shRNA-constructed with adeno-associated viral (AAV) serotype 8 (AAV8), by which a 65.8% reduction in mRNA and 62.7% reduction in protein of liver TRPC3 expression were attained (Fig. 3a and b). The tissue specificity test confirmed that only liver TRPC3 was significantly decreased in the measured tissues (Supplementary Fig. S4). Liver-specific TRPC3 loss exacerbated alcohol-induced liver injury, evidenced by the measurements of hematoxylin and eosin (H&E) staining and plasma levels of alanine transaminase (ALT) and aspartate transaminase (AST) (Fig. 3c–e). Caspase 3 activity assay indicated that TRPC3 knockdown aggravated alcohol consumption-induced apoptosis in liver samples (Fig. 3f). Further, proteins in apoptosis-related pathway, including apoptosis signal-regulating kinase 1 (ASK1) and c-Jun N-terminal kinase (JNK)/p38 MAPK, which have been reported implicating in ALD [21, 22], were determined in this study. Our data indicated that genetically silencing TRPC3 significantly upregulated alcohol-induced increase of p-ASK1, p-JNK, and p-p38 (Fig. 3g). We also observed consistent results in cultured hepatocytes (Supplementary Figs S5 and S6).

### TRPC3 knockdown deteriorates alcohol-induced hepatic steatosis

**Figure 4:**
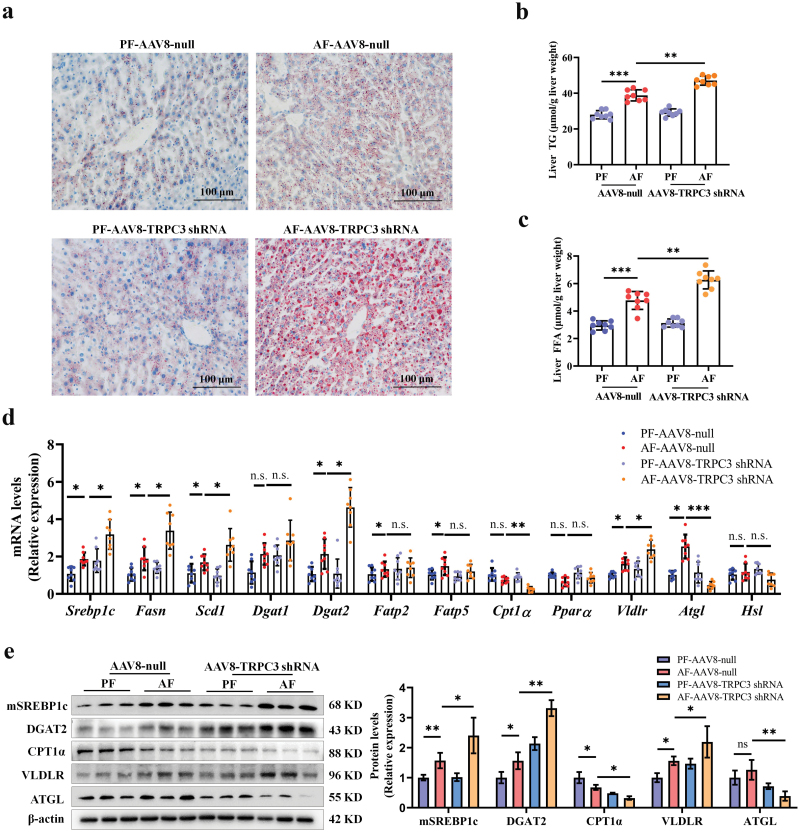
Liver-specific TRPC3 knockdown deteriorates alcohol-induced hepatic steatosis in mice. (a) Liver Oil red O staining. (b and c) Liver TG and FFA contents. (d and e) The mRNA and protein levels of lipid metabolizing enzymes in mouse liver. Protein band intensity was quantified by ImageJ. Data are presented as means ± SD (*n* = 6–8 for animals). ^*^P < 0.05, ^**^P < 0.01, ^***^P < 0.001 compared with corresponding control. n.s. represents no statistical difference.

Mice with liver-specific TRPC3 knockdown manifested mild fatty liver development compared to control mice, evidenced by Oil red O staining (Fig. 4a). Importantly, liver-specific TRPC3 loss exacerbated alcohol-induced triglyceride (TG) and free fatty acid (FFA) accumulation in the liver (Fig. 4b and c). In cultured hepatocytes, TRPC3 inhibition, by either genetic or pharmacological approach, aggravated intracellular lipid deposition induced by ethanol (Supplementary Fig. S7). Furthermore, in this study, we observed that TRPC3 knockdown was associated with increased expression of (i) SREBP-1c, which controls hepatic *de novo* lipogenesis; (ii) DGAT2, which controls TG synthesis; and (iii) VLDLR, which controls lipid uptake; and with decreased expression of (i) CPT1α, which controls FFA β-oxidation; (ii) ATGL (adipose triglyceride lipase), which catalyzes the hydrolysis of TGs in AF mouse liver (Fig. 4d and e). Additionally, loss of TRPC3 impaired mitochondrial function, evidenced by the down-regulated mitochondrial electron transport chain (ETC) subunits (MTCO1, CI-NDUFB8, and CII-SDHB) in AF mouse liver (Supplementary Fig. S8). Besides, genetic knockdown of TRPC3 enhanced mitochondrial polymerization, and reduced oxygen consumption rate (OCR) and mitochondrial membrane potential (MMP) in the presence of alcohol (Supplementary Figs S9–S11).

### TRPC3 loss promotes alcohol-associated hepatic inflammation and fibrosis

**Figure 5:**
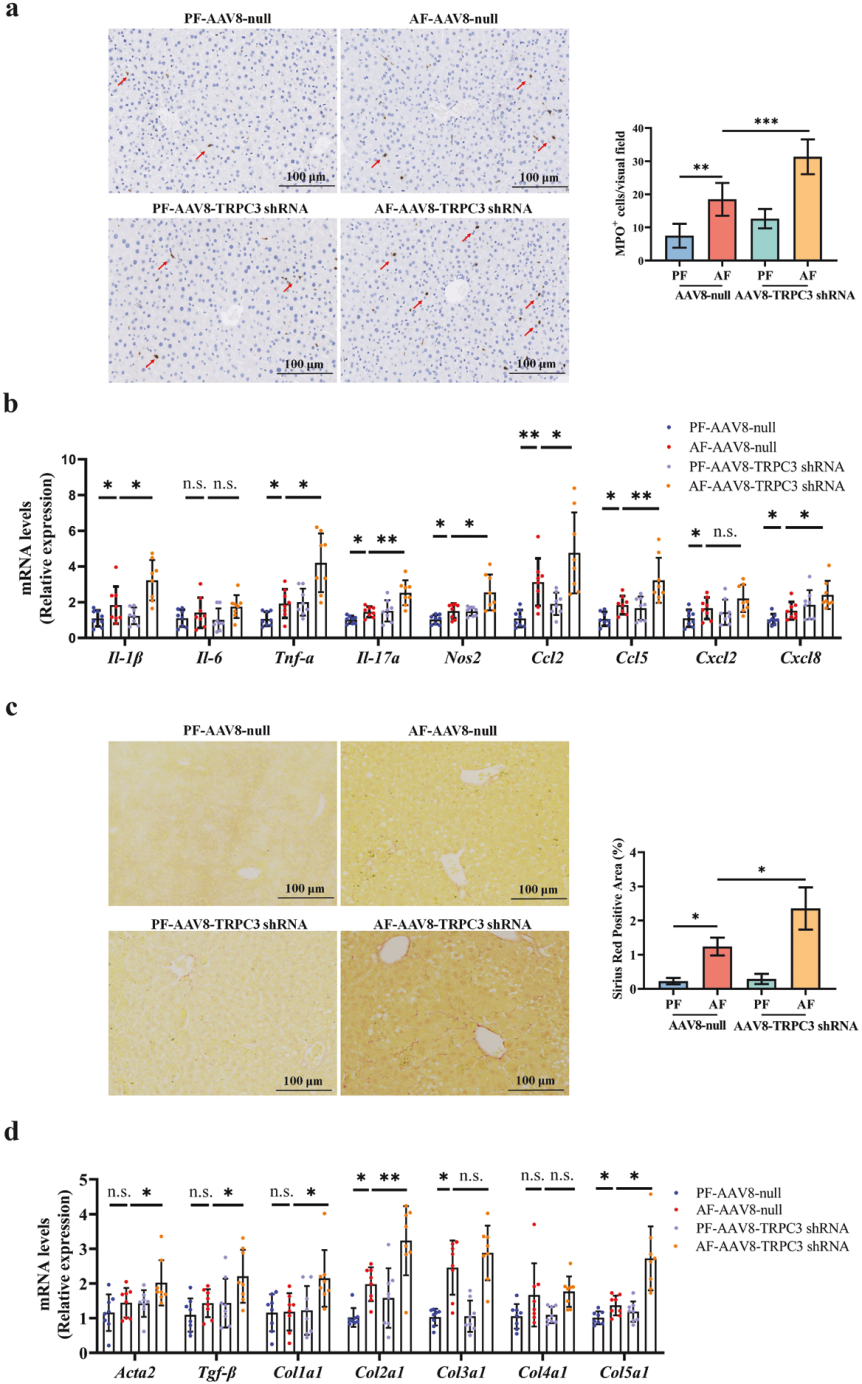
Liver-specific TRPC3 loss promotes alcohol-associated hepatic inflammation and early fibrosis-like change in mice. (a) Liver immunohistochemistry of myeloperoxidase (MPO) staining. (b) mRNA expression of *Il-1β*, *Il-6*, *Tnf-α*, *Il-17α, Nos2*, *Ccl2*, *Ccl5*, *Cxcl2*, and *Cxcl8* in mouse liver. (C) Liver Sirius red staining. (D) mRNA expression of *Acta2*, *Tgf-β*, *Col1a1*, *Col2a1*, *Col3a1*, *Col4a1*, and *Col5a1* in mouse liver. Data are presented as means ± SD (*n* = 4–8 for animals). ^*^P < 0.05, ^**^P < 0.01, ^***^P < 0.001 compared with corresponding control. n.s. represents no statistical difference.

Immune cell infiltration and increased pro-inflammatory cytokine production in the liver are hallmarks of ALD. Our data showed that TRPC3 loss significantly enhanced alcohol-induced neutrophil infiltration and transcriptionally upregulated pro-inflammatory cytokines, including *Il-1β*, *Tnf-α*, *Il-17α, Nos2, Ccl2, Ccl5, Cxcl2*, and *Cxcl8* (Fig. 5a and b). Additionally, alcohol-induced early fibrosis-like change in hepatic sinuses area was aggravated in liver-specific TRPC3 knockdown mice, along with the upregulation of *Acta2, Tgf-β, Col1a1, Col2a1*, and *Col5a1* mRNA expression (Fig. 5a and d).

### Lipolysis of adipose tissue is irrelevant to liver TRPC3-regulated ALD

The enhancement of lipolysis in adipose tissue has been reported to be implicated in alcohol-associated hepatic steatosis [23]. In our study, alcohol consumption lowered fat weight and fat weight to body weight ratio, as well as increased circulatory glycerol and FFA levels (Supplementary Fig. S12). However, TRPC3 knockdown did not enhance alcohol-stimulated lipolysis in adipose tissue (Supplementary Fig. S12), excluding the participation of lipolysis in liver TRPC3-regulated ALD.

### TRPC3 overexpression relieves alcohol-induced hepatic injury

**Figure 6:**
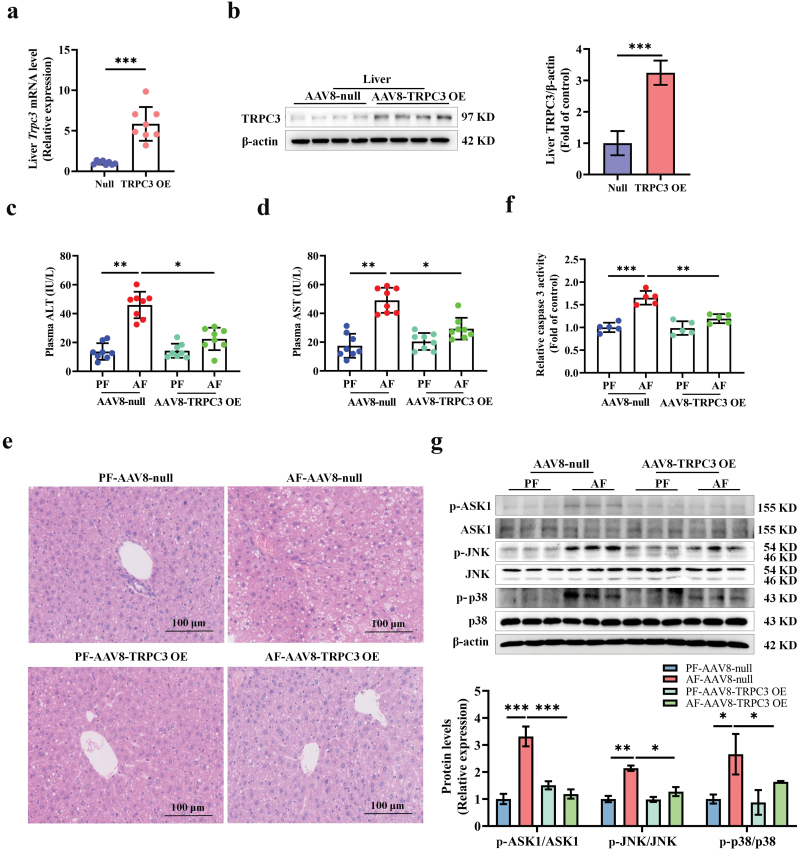
Liver-specific TRPC3 overexpression alleviates alcohol-induced hepatic injury in mice. Liver-specific TRPC3 overexpression mice were generated by caudal vein injection with AAV8 constructed vector containing TRPC3 sequence. Mice injected with null-vector were served as control. (a and b) TRPC3 mRNA and protein expressions in the liver of hepatocyte-specific TRPC3 overexpression mouse. (c and d) Plasma ALT and AST levels. (e) Liver H&E staining. (f) Caspase 3 activity in mouse liver. (g) Phosphorylated-ASK1, -JNK, and -p38 protein expressions were detected in mouse liver. Protein band intensity was quantified by ImageJ. Data are presented as means ± SD (*n* = 6–8 for animals). ^*^P < 0.05, ^**^P < 0.01, ^***^P < 0.001 compared with corresponding control.

To directly test the therapeutic potential of TRPC3 in ALD, liver-specific TRPC3 overexpression mice were generated via caudal vein injection with full-length TRPC3 sequence-constructed plasmid with AAV8. The overexpression efficiency was confirmed by western blot (Fig. 6a and b), and among the tissues tested, only liver TRPC3 was increased (Supplementary Fig. S13). Our data showed that TRPC3 overexpression significantly restored alcohol-induced adverse morphological changes in mouse liver (Fig. 6e) and the elevation of ALT and AST levels (Fig. 6c and d). Caspase 3 activity assay indicated that TRPC3 overexpression improved alcohol consumption-induced apoptosis in liver samples (Fig. 6f). Alcohol-induced increases of p-ASK1, p-JNK, and p-p38 were significantly reversed by TRPC3 overexpression (Fig. 6g). Consistent results were also observed in TRPC3 overexpressed hepatocytes (Supplementary Figs S14 and S15).

### TRPC3 overexpression alleviates alcohol-induced hepatic steatosis

**Figure 7:**
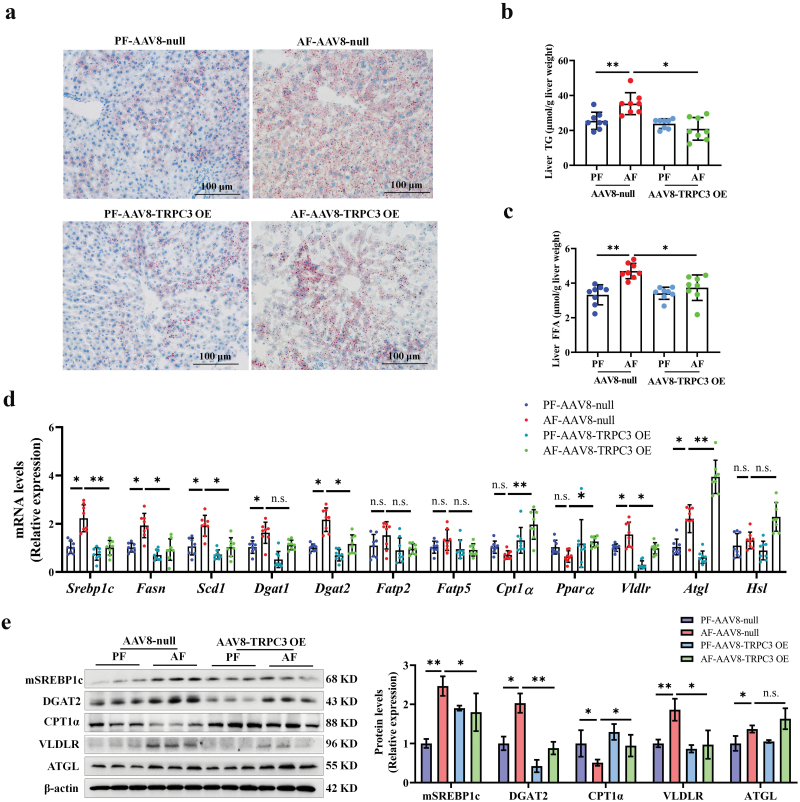
Liver-specific TRPC3 overexpression alleviates alcohol-induced hepatic steatosis in mice. (a) Liver Oil red O staining. (b and c) Liver TG and FFA contents. (d and e) The mRNA and protein levels of lipid metabolizing enzymes in mouse liver. Protein band intensity was quantified by ImageJ. Data are presented as means ± SD (*n* = 6–8 for animals). ^*^P < 0.05, ^**^P < 0.01, and ^***^P < 0.001 compared with corresponding control. n.s. represents no statistical difference.

Having established that TRPC3 deficiency is causally linked to alcohol-associated hepatic steatosis, we further explored whether overexpression of TRPC3 is sufficient to improve alcohol-induced hepatic steatosis in mice. The increase in liver fat due to chronic alcohol consumption was significantly ameliorated in mice with liver TRPC3 overexpression (Fig. 7a–c). TRPC3 restoration improved alcohol-triggered dysregulation of genes related to lipid metabolism (Fig. 7d and e). Additionally, TRPC3 overexpression restored the expression of oxidative phosphorylation (OXPHOS) machinery subunits (CI-NDUFB8 and CII-SDHB) in mouse liver mitochondria (Supplementary Fig. S16). In cultured hepatocytes, TRPC3 overexpression improved alcohol-reduced OCR (Supplementary Fig. S17).

### TRPC3 overexpression improves alcohol-induced hepatic inflammation and fibrosis

**Figure 8:**
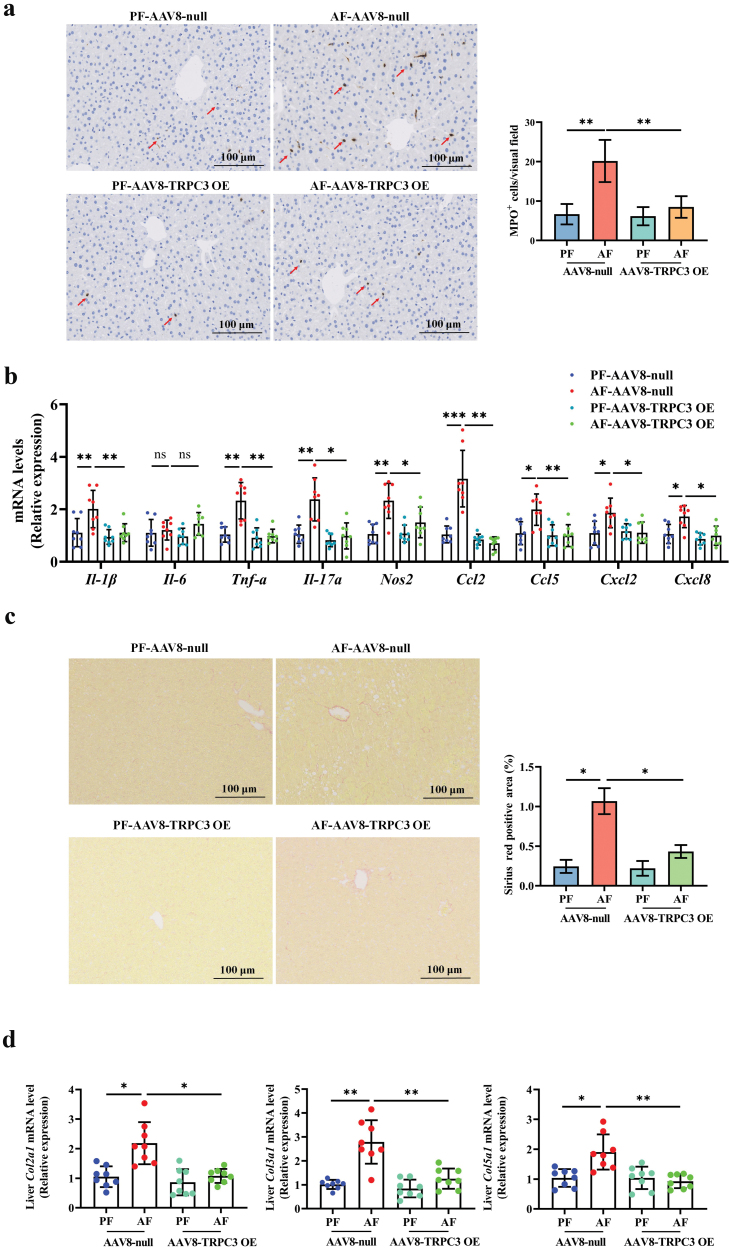
TRPC3 overexpression alleviates alcohol-induced inflammation and fibrosis in mice. (a) Liver immunohistochemistry of MPO staining. (b) mRNA expression of *Il-1β*, *Il-6*, *Tnf-α*, *Il-17α*, *Nos2*, *Ccl2*, *Ccl5*, *Cxcl2*, and *Cxcl8* in mouse liver. (c) Sirius red staining of liver samples. (d) mRNA expression of *Col2a1, Col3a1,* and *Col5a1* in mouse liver. Data are presented as means ± SD (*n* = 6–8 for animals). ^*^P < 0.05, ^**^P < 0.01, ^***^P < 0.001 compared with corresponding control. n.s. represents no statistical difference.

We further tested the effect of TRPC3 overexpression on alcohol-induced hepatic inflammation and fibrosis. Our data showed that TRPC3 overexpression significantly alleviated alcohol-induced neutrophil infiltration and transcriptionally upregulated pro-inflammatory cytokines, including *Il-1β*, *Tnf-α*, *Il-17α, Nos2, Ccl2, Ccl5, Cxcl2,* and *Cxcl8* (Fig. 8a and b). Additionally, alcohol-induced early fibrosis-like change in the hepatic sinuses area was rescued in liver-specific TRPC3 overexpression mice, along with the downregulation of *Col2a1*, *Col3a1*, and *Col5a1* mRNA expression (Fig. 8c and d).

### TRPC3 modulates AMPK in a Ca^2+^/CAMKK2-dependent manner in response to alcohol exposure

**Figure 9:**
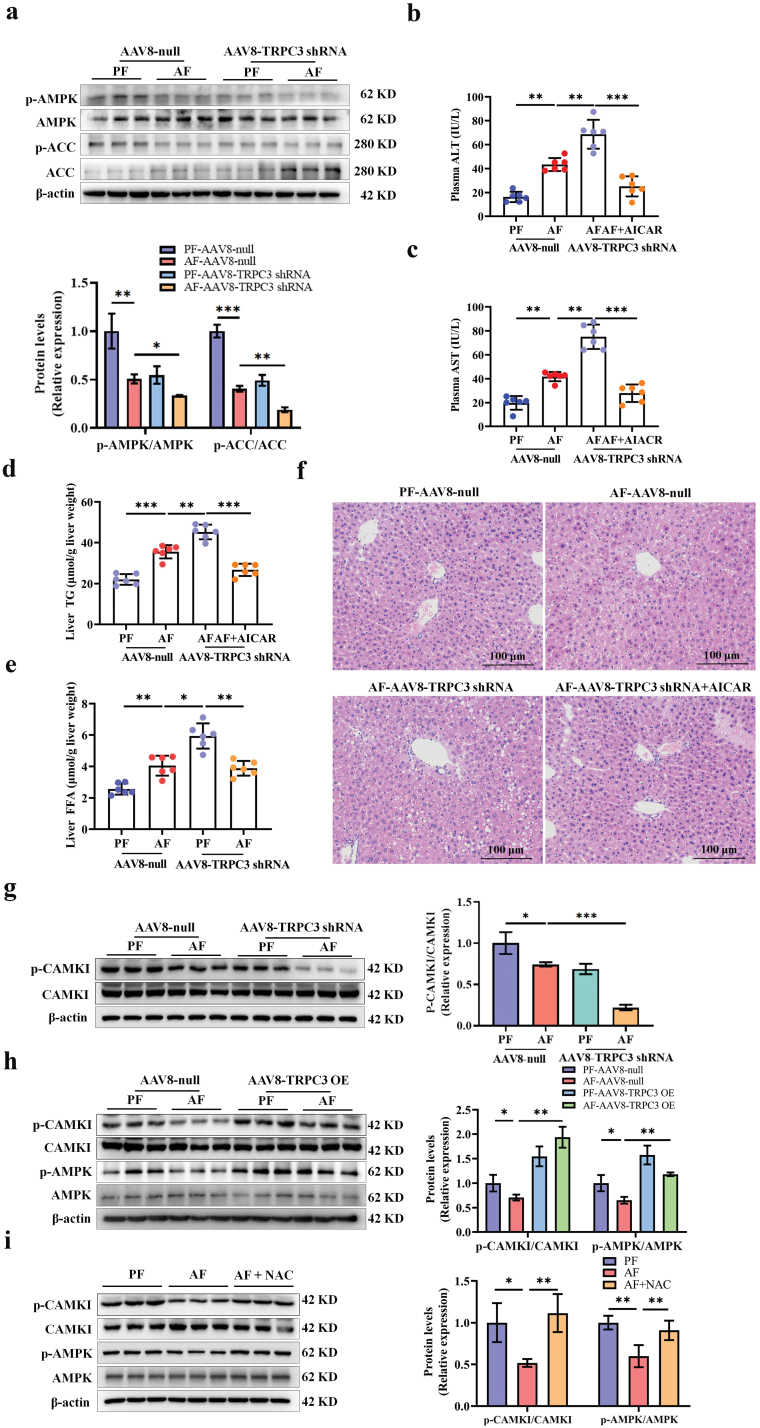
TRPC3 modulates AMPK in a Ca2^+^/CAMKK2-dependent manner in response to alcohol exposure. (a) Phosphorylated-AMPK and -ACC were detected in mouse liver samples. (b–f) Plasma ALT and AST levels (b and c), liver TG and FFA contents (d and e), and liver H&E staining (f) were determined in mice chronically fed alcohol and supplemented with AICAR (150 mg/kg body weight/day) after hepatic TRPC3 knockdown. (g) Phosphorylated-CAMKI level in the livers of hepatocyte-specific TRPC3 knockdown mice. (h) Phosphorylated-CAMKI and -AMPK in the livers of hepatocyte-specific TRPC3 overexpression mice. (i) NAC supplementation reversed phosphorylation levels of CAMKI and AMPK in chronic AF mice. Data are presented as means ± SD (*n* = 6–8 for animals). ^*^P < 0.05, ^**^P < 0.01, ^***^P < 0.001 compared with corresponding control. n.s. represents no statistical difference.

AMPK inhibition is positively associated with the abnormal expression of genes involved in lipid metabolism [24]. Suppressed AMPK activation contributes to hepatic steatosis development in ALD [25]. In an attempt to explore the mechanism by which TRPC3 knockdown aggravated hepatic steatosis in ALD, our data uncovered that TRPC3 deficiency was associated with AMPK suppression in mouse liver, evidenced by decreased protein abundance of p-AMPK and its down-stream target, p-ACC (Fig. 9a; Supplementary Fig. S18). AMPK activation by 5-aminoimidazole-4-carboxamide ribonucleotide (AICAR), a specific AMPK agonist, significantly attenuated TRPC3 knockdown-enhanced hepatic injury and lipid accumulation in chronic AF mice (Fig. 9b–f), implying that AMPK suppression contributes to TRPC3-mediated lipid abnormality. In addition, in cultured hepatocytes, AMPK inhibition, by either genetic or pharmacological approach, blocked TRPC3 overexpression-protected lipid deposition in response to alcohol exposure (Supplementary Fig. S19). Two classic protein kinases controlling AMPK activity, including liver kinase B1 (LKB1) and CAMKK2, were determined subsequently. Our results revealed that knocking down CAMKK2 rather than LKB1 blocked TRPC3 overexpression-protected TG accumulation under alcohol exposure (Supplementary Fig. S20). Phosphorylated-calmodulin-dependent protein kinase I (CaMKI), a well-known reporter of CaMKK2 activity [26], was evaluated in this study. Our data revealed that loss of TRPC3 deteriorated alcohol-inhibited CAMKI phosphorylation in both mouse liver and cultured hepatocytes (Fig. 9g; Supplementary Fig. S21). CAMKK2 activation by its special agonist (Methyl cinnamate) alleviated TRPC3 knockdown-enhanced lipid accumulation in the presence of alcohol (Supplementary Fig. S22). TRPC3 restoration blunted alcohol-induced suppression of CaMKK2 and AMPK (Fig. 9h). This phenomenon also occurred with the intervention of NAC, which reversed alcohol-triggered CaMKK2 and AMPK inhibition in both mouse liver and cultured hepatocytes (Fig. 9i; Supplementary Fig. S23). TRPC3 operates the transmembrane transport of cations, especially Ca^2+^ influx [27], which in turn stimulates CAMKK2 and AMPK activation [28]. Here, we observed that TRPC3 silence blunted thapsigargin-triggered Ca^2+^ influx (Supplementary Fig. S24). These results altogether suggest that the impairment of TRPC3-controlled Ca^2+^ influx plays a critical role in alcohol-induced hepatic steatosis development via inhibiting the CAMKK2/AMPK pathway.

## Discussion

**Figure 10:**
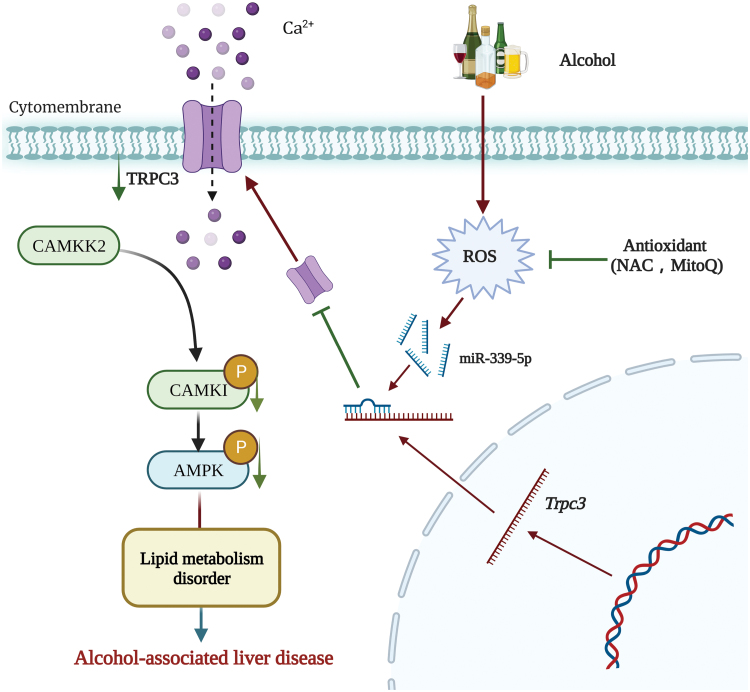
The proposed model of hepatic TRPC3-regulated ALD.

This study provides original evidence that hepatic TRPC3 reduction contributes to ALD pathogenesis. Mice with liver-specific TRPC3 silencing manifested mild fatty liver and exacerbated liver pathologies of ALD upon chronic alcohol consumption, whereas genetic replenishment of hepatic TRPC3 ameliorated ALD development. Our mechanistic investigations uncover that hepatic oxidative stress contributes to alcohol-induced TRPC3 loss associated with miR-339-5p upregulation, and via suppressing activation of the CaMKK2/AMPK pathway, TRPC3 downregulation leads to aggravation of alcohol-induced liver injury and dysregulation of gene expression related to liver lipid homeostasis, favoring fatty liver development (Fig. 10).

Accumulated evidence supports the involvement of calcium channel protein in the pathological process of liver diseases [29]. As a calcium-permeable non-selective cation channel, the transient receptor potential (TRP) channel superfamily is the most investigated one for its remarkable roles in various physiological and pathological states. In mammals, the TRP channel superfamily consists of TRPC, TRPM (melastatin), TRPV (vanilloid), TRPA (ankyrin), TRPP (polycystin), and TRPML (mucolipin) subfamilies [30]. TRP channels exhibit both pro- and anti-hepatic steatosis activity, evidenced by the observation that loss of TRPC5, TRPV1, and TRPM2 protected [31–33], whereas loss of TRPV4 aggravated hepatic steatosis [34]. To the best of our knowledge, no published data have ever evaluated the role of TRPCs in ALD.

TRPCs contain seven isoforms (TRPC1−7), among which, TRPC2 is a fake gene and is not expressed in human beings [35]. In this study, we report for the first time that (i) chronic alcohol consumption reduces hepatic TRPC3 expression without affecting other TRPCs; (ii) miR-339-5p contributes to TRPC3 reduction in response to alcohol-induced oxidative stress; (iii) TRPC3 loss aggravates alcohol-induced hepatic damage, steatosis, and mitochondrial subunit abundance; (iv) restoring hepatic TRPC3 arrests the pathological progression of alcohol-induced liver disease; and (v) CaMKK2/AMPK suppression contributes to TRPC3 loss-mediated hepatocyte injury and dysregulated lipid metabolism.

At present, the relationship between TRPC3 and ALD is entirely unknown. A recent study has reported that globally depleting TRPC3 neither attenuated nor worsened liver steatosis induced by choline-deficient, L-amino acid-defined, and high-fat diet [36]. However, endothelial-specific TRPC3 overexpression aggravated high-fat diet-induced hepatic steatosis compared with that in littermate controls [37]. This evidence suggests the tissue/cell specificity of TRPC3 in the regulation of liver lipid metabolism. The present study primarily focused on investigating the function of TRPC3 in hepatocytes from ALD mice, as our study demonstrated that hepatic TRPC3 knockdown aggravated, while hepatic TRPC3 recovery alleviated, lipid accumulation in mouse liver when being challenged by ethanol exposure. It is necessary to mention that the alcohol dosage (200 mmol/L) was selected according to several previous studies to establish a stable lipid deposition and/or cell damage model [17, 38, 39]. However, this dosage is much higher than the actual concentration in human blood that can be achieved by alcohol consumption, which cannot truly represent the reality of human body. Another limitation of this study is the lack of data from liver-specific TRPC3 knockout or transgenic mice *in vivo.* Besides hepatocytes, data from mouse liver single-cell sequencing showed that TRPC3 was also expressed in endothelial cells, stellate cells, hepatocytes, Kupffer cells, and T cells [40]. Further studies are necessary to identify the role of TRPC3 in other cell types in the liver from ALD mice.

The mechanisms implicated in alcohol-induced fatty liver development are multifactorial, among which AMPK, a central regulator of energy metabolism, plays an essential role in alcohol-induced fat accumulation in the liver [41]. Chronic alcohol consumption is associated with hepatic AMPK suppression [42], and AMPK activation protects against ALD development [42]. However, the exact mechanisms linking alcohol exposure and AMPK inhibition remain to be clarified. In line with previous studies, we observed a strong inhibition of hepatic AMPK in our mouse model of ALD. Notably, we identified that alcohol-induced TRPC3 loss contributes to AMPK suppression and subsequent lipid accumulation in the liver due to that (i) genetic knockdown of liver TRPC3 enhanced AMPK suppression by alcohol; (ii) TRPC3 overexpression alleviated alcohol-induced AMPK suppression; and (iii) AMPK activation rescued liver TRPC3 knockdown-aggravated ALD.

AMPK is mainly regulated by Ca^2+^-dependent CaMKK2 activation [43]. It has been documented that TRPC channels are required for Ca^2+^ influx-regulated CAMKK2/AMPK activation [44–47]. In this study, we confirmed that CaMKK2 plays a mechanistic role in TRPC3-regulated AMPK activation, based on the observations that (i) TRPC3 loss aggravated alcohol-induced CaMKK2 activity suppression and (ii) TRPC3 overexpression attenuated alcohol-induced CaMKK2 inhibition. Emerging evidence has revealed that calcium ion channel dysfunction contributes to the pathological development of liver diseases, including ALD [48, 49]. Impaired Ca^2+^ influx and CaMKK2 inactivation have been observed in ALD previously [50]. TRPC3 loss/inhibition disrupts hepatic extracellular Ca^2+^ influx [51]. Here, we observed that TRPC3 knockdown blunted thapsigargin-induced hepatic extracellular Ca^2+^ influx, implicating that TRPC3 reduction contributes to alcohol-disrupted hepatic Ca^2+^ influx. These evidence has collaboratively implied that a Ca^2+^/CaMKK2-dependent pathway contributes to the regulation of AMPK by TRPC3. We have also tried to confirm these findings in perfusion-isolated intact primary hepatocytes from either chronic alcohol-exposed or TRPC3 knockdown and TRPC3 overexpressed mice. However, most isolated cells lost their response to stimuli-induced Ca^2+^ influx, which failed to objectively reflect the real situation *in vivo*, and this is a limitation of this study. Additionally, as a non-selective cation channel, TRPCs also control the mobility of other cations, including monovalent (Na^+^, K^+^, Cs^+^) and divalent (Mg^2+^) cations [52]. In further studies, it will be interesting to know whether those cations are involved in TRPC3-regulated lipid metabolism in ALD.

We further investigated the mechanism(s) linking alcohol consumption to TRPC3 reduction. Changes in liver miRNA expression are a significant pathological mechanism in the development of ALD [53]. To the best of our knowledge, limited study has reported the regulation of miRNA on TRPC3 in ALD. Through bioinformatics analysis and validation, we found that miR-339-5p is an upstream regulator involved in the TRPC3 reduction in ALD. In support of this, the inhibitory regulation of miR-339-5p on TRPC3 has also been observed in *SKOV3* and *HeyA8* cells [54]. Further, we observed that antioxidant supplementation alleviated chronic alcohol consumption-induced TRPC3 reduction in mouse liver. In support of us, it has been reported that oxidative stress also decreased TRPC3 in B lymphoblast cells [55]. Meanwhile, antioxidants suppressed the expression of miR-339-5p, suggesting that oxidative stress might play a key role in the regulation of miR-339-5p and TRPC3. Oxidative stress and AMPK inactivation are crucial pathological factors in the development of ALD [25]. However, the existing evidence did not fill the gap between oxidative stress induction and AMPK suppression. This study provides the first line of evidence that the oxidative stress-elicited miR-339-5p/TRPC3 axis contributes to AMPK inactivation in ALD via a Ca^2+^/CaMKK2-dependent pathway.

In conclusion, we provide original evidence that hepatic TRPC3 is required to protect against ALD, and hepatic TRPC3 reduction promotes the pathological progress of ALD. Our study suggests that restoring hepatic TRPC3 can be a potential therapeutic choice for the treatment of ALD.

## Materials and methods

### Human liver samples

Human liver samples from both healthy and AH individuals were generously provided by Dr. Zhaoli Sun from Johns Hopkins University School of Medicine. The detailed information has been described previously [56].

### Animals

Animal procedures were approved by the Institutional Animal Care and Use Committee of Zhejiang Chinese Medical University (ZSLL-2017-150). All mice were housed on a 12 h light/12 h dark cycle at 23 ± 2°Cwith 55 ± 5% relative humidity. Male C57BL/6J mice were fed with Lieber-De Carli alcohol liquid diet and Lieber-De Carli plus binge as described previously [57, 58]. Liver-specific TRPC3 knockdown or overexpression mice were generated by lateral tail vein injection with recombinant AAV8 gene transfer vectors bearing a hepatocyte-specific promoter in combination with either mouse TRPC3 shRNA sequence (AAV8-TRPC3 KD) or TRPC3 full-length sequence (AAV8-TRPC3 OE). NAC (40 mg/kg body weight/day) was given by gavage. MitoQ (5 mg/kg body weight/day) and AICAR (150 mg/kg body weight/day) were given by intraperitoneal injection. The detailed protocols were shown in the Supplementary Materials.

### Biochemical analysis

Plasma was separated from blood after centrifugation with 3000 rpm at 4°C for 15 min and then kept at −80°C until use. Plasma levels of ALT and AST were determined by ALT and AST determination kit (Nanjing Jiancheng Bio Co., Nanjing, China).

### Lipid content assay

TGs in liver samples were assayed using a TG kit (Applygen, Beijing, China) according to the manufacturer’s recommended protocol. Liver FFA content was measured by a commercial FFA assay kit (Nanjing Jiancheng Bio Co., Nanjing, China).

### Histological staining

For H&E staining, liver tissue samples were fixed in a 10% paraformaldehyde solution and further embedded in paraffin. Liver sections (4 μm) were deparaffinized in xylene and rehydrated through a series of decreasing ethanol concentrations. Sections were stained with H&E using a staining kit from G-Clone (Beijing, China). For Oil red O stain, liver tissues were embedded in the Tissue-Tek OCT compound (Sakura, Tokyo, Japan). Frozen sections, 8-μm thick, were subjected to Oil red O staining according to the instructions of the Oil red O stain kit (Solarbio, Beijing, China). For Sirius red staining, paraffin sections (4 μm) were hydrated and stained in Sirius red solution (G-Clone, Beijing, China) at room temperature for 1 h. Images were captured by Zeiss Axio Observer A1 inverted microscope (Oberkochen, Germany).

### Immunohistochemistry

Liver tissue paraffin sections were incubated with 3% hydrogen peroxide for 10 min to inactivate endogenous peroxidases and with normal serum (from the same species producing the secondary antibody) for 20 min. Then, the tissue sections were incubated with primary antibodies (Supplementary Table S2) at 4°C overnight, followed by incubation with the corresponding Dako EnVision+ System HRP Labelled Polymer Anti-Rabbit secondary antibody (Agilent, Santa Clara, CA) at room temperature for 30 min. Visualization was conducted with diaminobenzidine and hydrogen peroxide. Images were captured by Zeiss Axio Observer A1 inverted microscope (Oberkochen, Germany).

### Cell culture

Primary mouse hepatocytes were isolated as reported previously [16, 59]. Briefly, mice were anesthetized with pentobarbital (30 mg/kg body weight, intraperitoneally). Livers were firstly perfused with ice Hank’s Balanced Salt Solution (Monad, Wuhan, China) via the portal vein and followed by digestion for 15 min at 37°C in digestion buffer (RPMI-1640 containing 1% fetal bovine serum (FBS, Biological Industries, ISR), 0.1 mg/mL DNase-I (Sigma-Aldrich, St. Louis, MO), 0.2 mg/mL Collagenase IV (Sigma-Aldrich, St. Louis, MO), and 0.8 mg/mL Dispase II (Sigma-Aldrich, St. Louis, MO). After digestion, dissociated cells were collected and filtered through a 100 µm cell strainer (BD Biosciences, San Jose, CA) followed by centrifuge at 50 g for 3 min at 4°C. Pellets were suspended with 20 mL 40% ice-cold percoll (GE Healthcare, Bensalem, PA) and centrifuged at 180 g for 7 min at 4°C. After this step, primary hepatocytes were isolated. AML-12 and HEK293T cell lines were obtained from the American Type Culture Collection (ATCC, Manassas, VA). AML-12 cells were cultured in DMEM/F-12 containing 10% (v/v) FBS, 5 mg/mL insulin (Solarbio, Beijing, China), 5 μg/mL transferrin (Solarbio, Beijing, China), 5 ng/mL selenium (Sigma-Aldrich, St. Louis, MO), 40 ng/mL dexamethasone (Solarbio, Beijing, China); HEK293T cells were cultured in DMEM containing 10% (v/v) FBS. All cell lines were cultured at 37°C in 5% CO_2_, 95% air-humidified atmosphere.

### Luciferase reporter gene assay

Wild-type (WT) TRPC3 3ʹ-UTR and mutant-type (MUT) TRPC3 3ʹ-UTR sequences were imported into psiCHEK2.0 vectors (Thermo Fisher Scientific, Shanghai, China) to construct TRPC3-WT and TRPC3-MUT plasmids. WT or MUT TRPC3 3ʹ-UTR and miR-339-5p mimic/inhibitor were co-transfected into HEK293T cells by Lipofectamine 3000 reagent. After 24 h, the cells were obtained, and firefly and *Renilla* luciferase activities were measured sequentially with the dual luciferase assay kit (GenePharma, Shanghai, China). The activities were normalized with *Renilla* luciferase activities and expressed in relative luciferase activity units.

### Quantitative real-time polymerase chain reaction (qRT-PCR)

Total RNA was isolated from the tested tissues or cultured cells using Trizol reagent (Invitrogen, Carlsbad, CA). Extracted RNA was then transcribed into cDNA by reverse transcription reagent kits (Monad, Wuhan, China) according to the manufacturer’s protocol. The primers were synthesized by Sangon Biotech Co. Ltd (Shanghai, China), and primer sequences are shown in Supplementary Table S1. The abundance of miRNA and mRNA was normalized to U6 snRNA and 18s rRNA, respectively.

### Western blot analysis

Western blot was performed as described previously [57]. In brief, liver tissues or cultured cells were lysed with RIPA buffer (Boster Biological Technology, Wuhan, China) supplemented with protease and phosphatase inhibitors (Sigma-Aldrich, St. Louis, MO). The protein samples were loaded into SDS-PAGE gels and transferred to PVDF membranes (Millipore, Bedford, MA). The membranes were blocked with 5% fat-free milk in TBST (Servicebio, Wuhan, China) and reacted with primary antibodies at 4°C for 12–16 h. After washing with TBST, the membranes were incubated with secondary antibodies for 1 h at room temperature. Finally, the immunoreactivity of protein expression was visualized with an electrogenerated chemiluminescence kit (Vazyme, Nanjing, China). The immunoblots were quantified by measuring the density of each band with ImageJ Software. The antibodies are listed in Supplementary Table S2.

### Statistical analysis

The statistical analysis was performed with GraphPad Prism (GraphPad Software 8.0.1). Data are presented as the mean ± standard deviation (SD). One-way ANOVA followed by a post hoc test with Fisher’s least significant difference was employed for multi-group comparison. A comparison between the two groups was conducted using Student’s *t*-test. All *P*-values were two-tailed, and a *P*-value < 0.05 was considered significant for all statistical analyses.

## Supplementary Material

load050_suppl_Supplementary_Figures_S1-S24_Tables_1-2

## Data Availability

All study data are included in the article and/or Supplementary Materials. Materials are available upon request.
