# Supplementary material for: Hepatic TRPC3 loss contributes to chronic alcohol consumption-induced hepatic steatosis and liver injury in mice
Source: Life Metab. 2023 Dec 18;3(1):load050. doi: 10.1093/lifemeta/load050 (PMC11749259; doi:10.1093/lifemeta/load050)
Supplement: load050_suppl_Supplementary_Figures_S1-S24_Tables_1-2 [file load050_suppl_Supplementary_Figures_S1-S24_Tables_1-2.docx]

**Supplementary Material**

**Hepatic TRPC3 loss contributes to chronic alcohol consumption-induced hepatic steatosis and liver injury in mice**Qinchao Ding^1,2,#^, Rui Guo^1,#^, Liuyi Hao^1,#^, Qing Song^1,4^, Ai Fu^3^, Shanglei Lai^3^, Tiantian Xu^1^, Hui Zhuge^3^, Kaixin Chang^3^, Yanli Chen^3^, Haibing Wei^3^, Daxi Ren^2^, Zhaoli Sun^5^, Zhenyuan Song^4^, Xiaobing Dou^3,*^, Songtao Li^1,*^

^1^School of Public Health, Zhejiang Chinese Medical University, Hangzhou, Zhejiang 310053, China

^2^College of Animal Science, Zhejiang University, Hangzhou, Zhejiang 310053, China

^3^School of Life Science, Zhejiang Chinese Medical University, Hangzhou, Zhejiang 310053, China

^4^Department of Kinesiology and Nutrition, University of Illinois at Chicago, Chicago, IL 60612, USA

^5^Department of Surgery, Johns Hopkins University School of Medicine, Baltimore, MD 21205, USA

^#^These authors contributed equally to this paper.

* Corresponding author. S.L., School of Public Health, Zhejiang Chinese Medical University, Hangzhou, Zhejiang 310053, China. E-mail: [lisongtao@zcmu.edu.cn](mailto:lisongtao@zcmu.edu.cn); X.D., School of Life Science, Zhejiang Chinese Medical University, Hangzhou, Zhejiang 310053, China. E-mail: [xbdou77@163.com](mailto:xbdou77@163.com)

**Contents list**

Supplementary Materials and Methods………………………………………………. 4

Supplementary Figure S1…………………………………………………………….9

Supplementary Figure S2…………………………………………………………….10

Supplementary Figure S3…………………………………………………………….11

Supplementary Figure S4…………………………………………………………….12

Supplementary Figure S5…………………………………………………………….13

Supplementary Figure S6…………………………………………………………….14

Supplementary Figure S7…………………………………………………………….15

Supplementary Figure S8…………………………………………………………….16

Supplementary Figure S9…………………………………………………………….17

Supplementary Figure S10…………………………………………………………...18

Supplementary Figure S11…………………………………………………………...19

Supplementary Figure S12…………………………………………………………...20

Supplementary Figure S13…………………………………………………………...21

Supplementary Figure S14…………………………………………………………...22

Supplementary Figure S15…………………………………………………………...23

Supplementary Figure S16…………………………………………………………...24

Supplementary Figure S17…………………………………………………………...25

Supplementary Figure S18…………………………………………………………...26

Supplementary Figure S19…………………………………………………………...27

Supplementary Figure S20…………………………………………………………...28

Supplementary Figure S21…………………………………………………………...29

Supplementary Figure S22…………………………………………………………...30

Supplementary Figure S23…………………………………………………………...31

Supplementary Figure S24…………………………………………………………...32

Supplementary Table S1………………………………………………………….…33

Supplementary Table S2……………………………………………….……………36

Supplementary Reference………………………………………………...………...38

**Supplementary materials and methods**

***Chemicals***

Ethanol and N-acetylcysteine (NAC) were purchased from Sigma-Aldrich (St. Louis, MO); Pyr3 (a Transient receptor potential cation channel subfamily C member 3 (TRPC3) channel blocker), 5-aminoimidazole-4-carboxamide ribonucleotide (AICAR), Compound C, ML385 (an Nrf2 inhibitor), and **mitoquinone (**MitoQ) were obtained from MedChemExpress (Shanghai, China); SP600125, U0126, SB203580, and Ceapin-A7 were purchased from Selleck Chemicals (Shanghai, China); GSK2606414 (an ATP-competitive potent protein kinase R (PKR)-like ER kinase (PERK) inhibitor), STF083010 (an inositol requiring-enzyme 1α (IRE1α) RNase inhibitor,), and PX-478 (hypoxia-inducible factor-1α (HIF-1α) inhibitor) were purchased from APExBIO (Houston, TX); Methyl cinnamate was purchased from GlpBio (Shanghai, China);

***Animals***

Animal procedures were approved by the Institutional Animal Care and Use Committee of Zhejiang Chinese Medical University (ZSLL-2017-150). All mice were housed on a 12-h light-dark cycle at 23 ± 2℃ with 55 ± 5% relative humidity. All the interventions were performed after one week of environmental acclimatization. Food intake and body weight were recorded daily and weekly, respectively. At the end of the experiments, mice were sacrificed under pentobarbital solution (30 mg/kg body weight, intraperitoneally) after 4 h fasting. Plasma and tissue samples were collected for further analysis.

Liver-specific TRPC3 knockdown or overexpression mice were generated by lateral tail vein injection with recombinant adeno-associated viral (AAV) serotype 8 gene transfer vectors bearing a hepatocyte-specific promoter combination with mouse TRPC3 shRNA sequence (AAV8-TRPC3 shRNA) or TRPC3 full-length sequence (AAV8-TRPC3 OE) (HanBio Technology Co. Ltd., Shanghai, China). AAV8 vectors were administered by tail vein injection at a dose of 1 × 10^12^ viral titer/mL in a total volume of 100 μL/mice one week before alcohol feeding.
 Lieber-De Carli ALD model was established as described previously ^1^. Mice were fed with the Lieber-De Carli alcohol liquid diet (alcohol-fed; AF) or isocaloric maltose dextrin control liquid diet (pair-fed; PF) for four weeks. There are two groups in total (*n* = 8 mice/group), and animals were fed for 4 weeks. In the first week, mice were given a modified Lieber DeCarli liquid diet (Trophic Animal Feed High-Tech Co., Ltd. Nantong, China) without alcohol for adaption to the liquid diet for 3 days, then the AF group was given Lieber DeCarli liquid diet containing 1% ethanol for 2 days, 2% ethanol for 2 days where the ethanol content (%, v/v) in the diet was 4% for the second week and was gradually increased by 1% every week, reaching 6% in the last week. The amount of food given to the PF mice was the same as the AF mice consumed on the previous day. NAC was administrated by gavage at a dose of 40 mg/kg body weight/day. MitoQ was intervened by intraperitoneal injection at a dose of 5 mg/kg body weight/day. AICAR was intervened by intraperitoneal injection at a dose of 150 mg/kg body weight/day.

Lieber-De Carli plus single binge ALD model was established based on Lieber-De Carli ALD model. After a 4-week Lieber-De Carli alcohol liquid diet feeding, AF mice were administrated with one dose of ethanol (4 g/kg) by gavage, and PF mice were given isocaloric maltose dextrin. Samples were collected 4 h after the gavage.

**Cell culture**

AML-12, a non-transformed mouse hepatocyte cell line was obtained from the American Type Culture Collection (ATCC, Manassas, VA). AML-12 cells were cultured in DMEM/F-12 containing 10% (v/v) FBS, 5 mg/mL insulin (Solarbio, Beijing, China), 5 μg/mL transferrin (Solarbio, Beijing, China), 5 ng/mL selenium (Sigma-Aldrich, St. Louis, MO), 40 ng/mL dexamethasone (Solarbio, Beijing, China), 100 U/mL penicillin, 100 μg/mL streptomycin, at 37°C in a humidified atmosphere of 5% CO_2_ and 95% air.

VL-17A, a HepG2-based reformed cell line, was conducted according to previous study ^2,3^. HepG2 cell line was obtained from the Cell bank of the Chinese Academy of Sciences (Shanghai, China). HepG2 cells were stably transfected with both cytochrome P450 2E1 (CYP2E1) and alcohol dehydrogenase (ADH) to construct VL-17A cell line. VL-17A cells were cultured in DMEM (Gibco, Waltham, MA) supplemented with 10% FBS and 100 U/mL penicillin/streptomycin at 37°C in a humidified atmosphere of 5% CO_2_ and 95% air.

***RNA interference***

Cultured cells were transfected with TRPC3 siRNA, AMP-activated protein kinase (AMPK) α1 siRNA, **Ca^2+^/calmodulin-dependent protein kinase kinase 2 (**CAMKK2) siRNA, and LKB1 siRNA (Santa Cruz Biotechnology, Santa Cruz, CA) using Lipofectamine 3000 according to the manufacturer’s instructions. In the control group, cells were transfected with scramble siRNA (Santa Cruz Biotechnology, Santa Cruz, CA).

***Cellular TRPC3 overexpression***

TRPC3 overexpression lentivirus (Vector: pLV-TBG-mTRPC3-CMV-T2A-Puro) and control overexpression lentivirus (Vector: pLV-CMV-T2A-Puro) were constructed by Cyagen Biosciences Inc. (Guangzhou, China). Cultured cells were transfected with lentivirus (MOI = 1) using polybrene (5 μg/mL). Transfection efficiency is shown in Supplementary Fig. S18.

***Oxygen consumption rate (OCR) measurement***

OCR was measured with an XF96 Extracellular Flux Analyzer (Agilent Technologies, Santa Clara, CA) and the Seahorse XF Cell Mito Stress Test Kit (Agilent Technologies, Santa Clara, CA) as described previously ^4^. Briefly, cells were incubated in XF Assay Medium supplemented with 25 mmol/L glucose, 1 mmol/L pyruvate, and 2 mmol/L glutamine. Cells were equilibrated in a CO_2_-free incubator at 37°C for 1 h. Chemicals, including 2 µmol/L oligomycin, 2 µmol/L carbonylcyanide p-trifluoromethoxyphenylhydrazone (FCCP), and 1 µmol/L rotenone plus 1 µmol/L antimycin A, were added at the indicated time-point. OCR value was normalized by cell number.

***Mitochondrial morphology measurement***

Mitochondrial morphology was observed using MitoTracker staining. After the treatment, cells were incubated with 200 nmol/L Mito-Tracker Red CMXRos (Beyotime, Shanghai, China) for 30 min at 37°C. Images were captured with a confocal microscope (ZEISS, Oberkochen, Germany).

***Mitochondrial membrane potential (MMP)***

MMP was measured by a JC-1 fluorescent probe (Beyotime, Shanghai, China). After the indicated treatment, cells were incubated with JC-1 dye (10 μg/mL in medium) for 1 h at 37°C. Then cells were washed with PBS 3 times. Images were obtained by using fluorescence microscopy (ZEISS, Oberkochen, Germany) acquisition.

***Calcium influx***

A calcium influx test was performed according to a previous study ^5^. Briefly, cells were incubated in Hank’s balanced salt solution (HBSS) (Monad, Wuhan, China) containing 5 μmol/L Fluo-4 AM (Beyotime, Shanghai, China) for 30 min at 37°C, the solution was replaced with fresh HBSS, and then cells were incubated under the microscope for at least 10 min before observation to allow temperature stabilization to 36 ± 0.5 °C. The fluorescence intensity of Fluo-4 AM was continuously imaged at 1-s intervals under 488 nm excitation and 525 nm emission wavelength settings using confocal microscopy (ZEISS, Oberkochen, Germany). Thapsigargin (2 μmol/L) was added to induce calcium influx after 30 s baseline calcium measurement, during which Fluo-4 AM fluorescence was recorded every second, and the fluorescence intensity was analyzed by ZEN confocal software.

**Supplementary figures**

**Supplementary Figure S1**

**
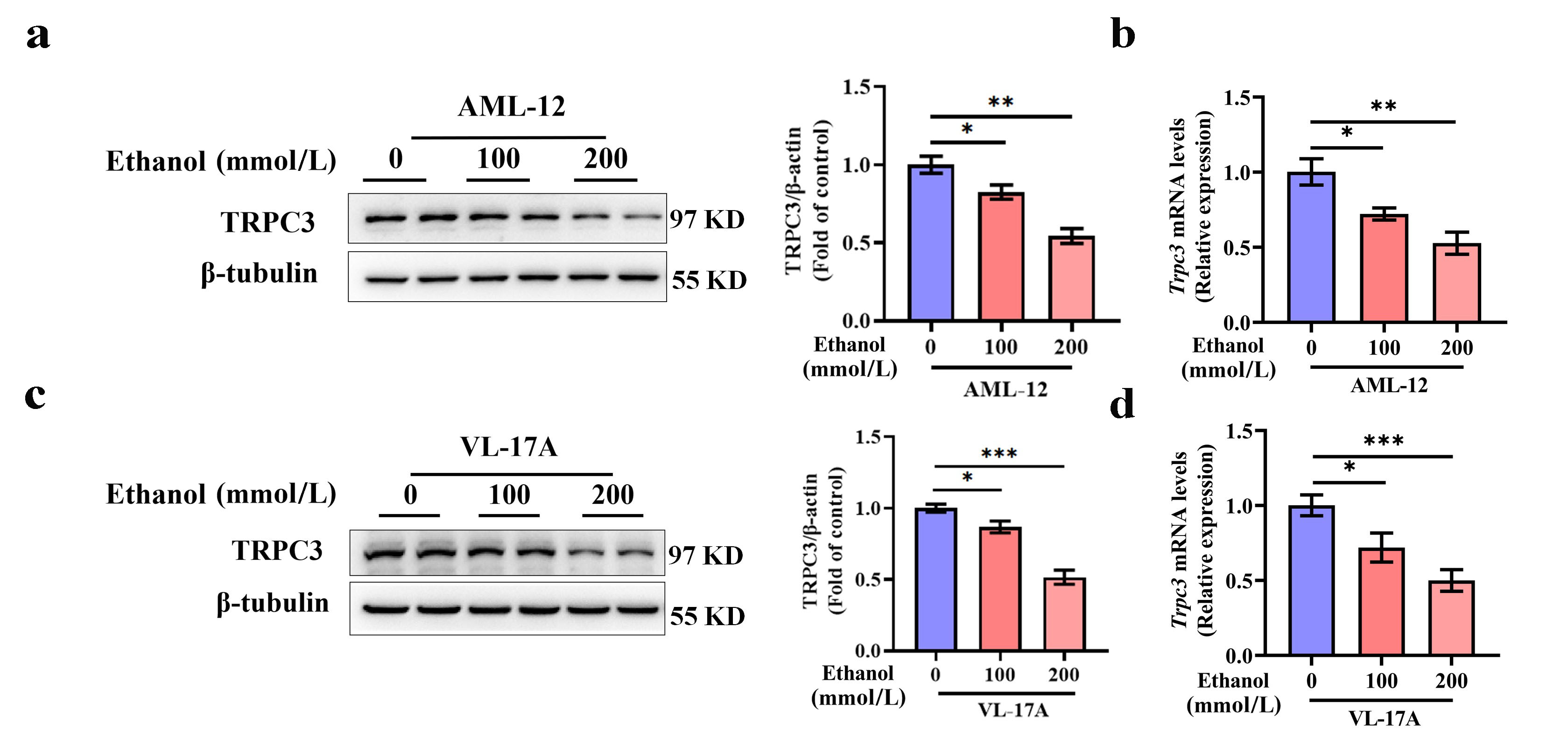
**

**Supplementary Figure 1** Alcohol exposure reduces protein and mRNA expression of TRPC3. AML-12 cells (a and b) and VL-17A cells (c and d) were treated with different doses of ethanol (100 and 200 mmol/L) for 48 h, respectively. Western blot was performed to analyze the expressions of TRPC3. Protein band intensity was quantified by ImageJ. qRT-PCR was performed to analyze the *Trpc3* mRNA level. Data are presented as means ± SD (*n* = 4). ^*^*P* < 0.05, ^**^*P* < 0.01, ^***^*P* < 0.001 compared with corresponding control.

**Supplementary Figure S2**


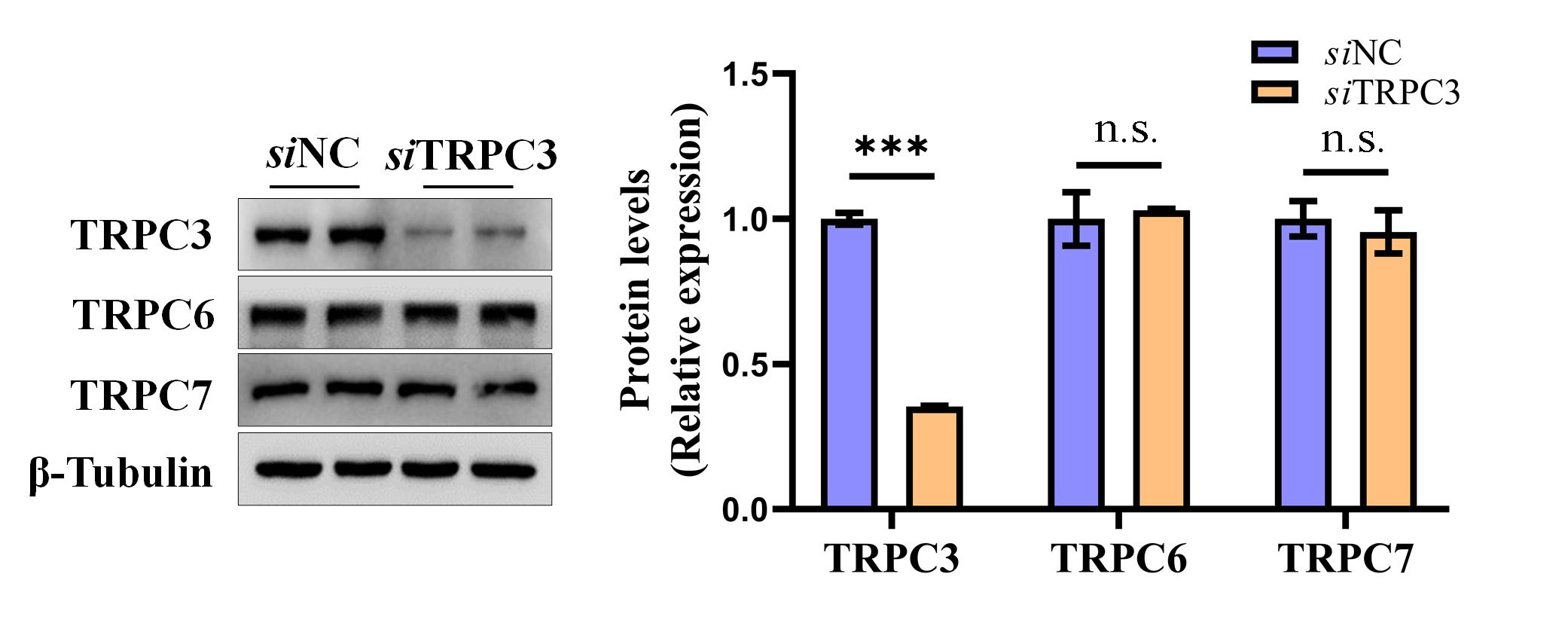


**Supplementary Figure 2** The specificity of TRPC3 antibody was confirmed by TRPC3 knockdown experiment. AML-12 cells were transfected with scramble siRNA (*si*NC) or TRPC3 siRNA (*si*TRPC3) for 48 h. Western blot was performed to analyze the expressions of TRPC3, TRPC6, and TRPC7. Protein band intensity was quantified by ImageJ. Data are presented as means ± SD (*n* = 4). ^***^*P* < 0.001 compared with corresponding control. n.s. represents no statistical difference.

**Supplementary Figure S3**

**
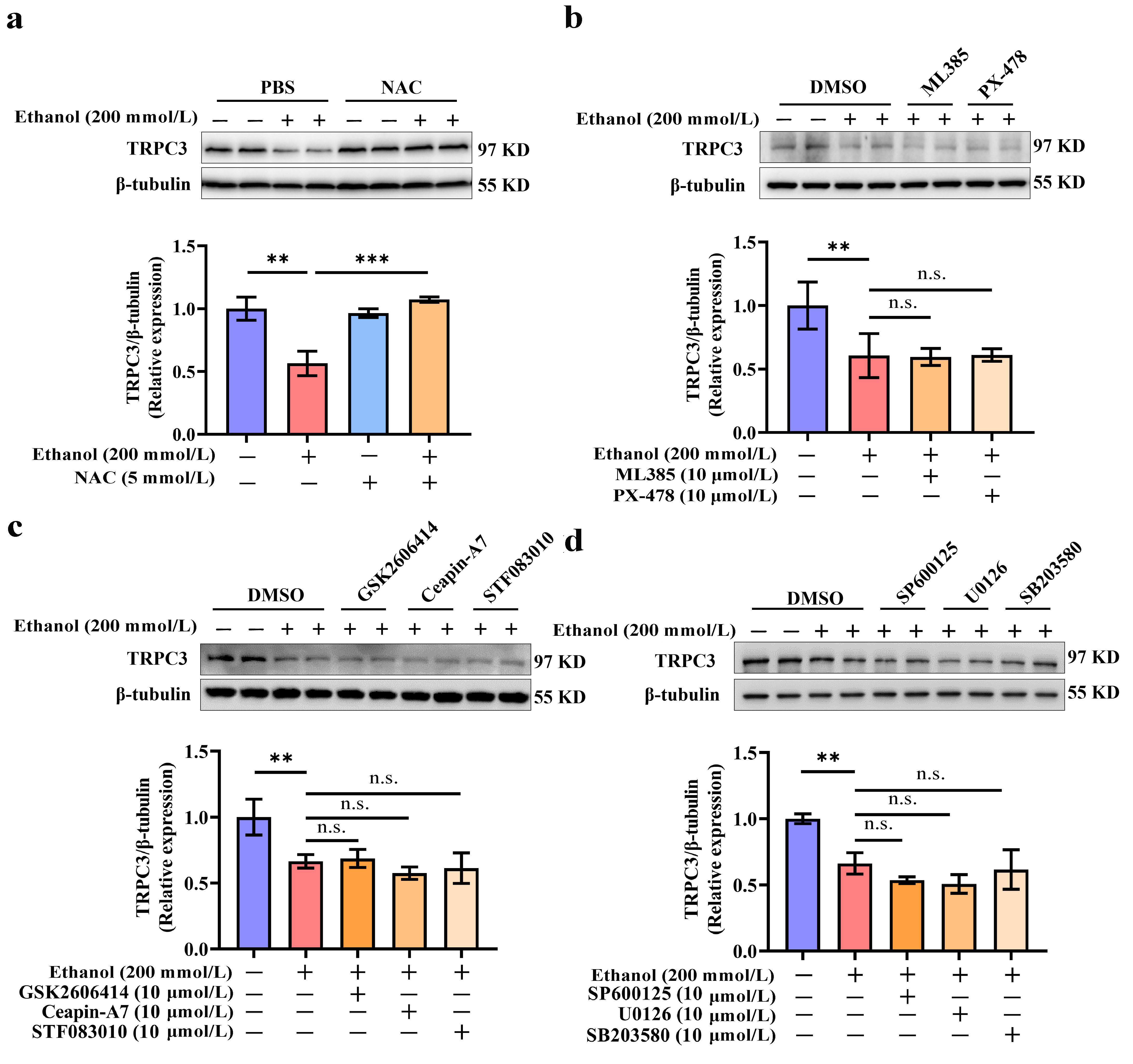
**

**Supplementary Figure S3** Oxidative stress decreases TRPC3 in ALD. (a−d) The chemical screening study identifies that ROS inhibitors restore TRPC3 downregulation with ethanol intervention. AML12 cells were treated with ethanol (200 mmol/L) for 48 h with or without a 2 h pretreatment with NAC (5 mmol/L), PERK inhibitor (GSK2606414, 20 μmol/L), activating transcription factor (ATF6) inhibitor (Ceapin-A7, 10 μmol/L), IRE1a inhibitor (STF083010, 40 μmol/L), Nrf2 inhibitor (ML385, 10 μmol/L), Hif-1a inhibitor (PX-478, 10 μmol/L), c-Jun N-terminal kinase (JNK) inhibitor (SP600125, 20 μmol/L), extracellular signal-regulated kinases (ERK) 1/2 inhibitor (U0126, 20 μmol/L) and p38 inhibitor (SB203580, 20 μmol/L), and TRPC3 protein expression was detected. Protein band intensity was quantified by ImageJ. Data are presented as means ± SD (*n* = 4). ^**^*P* < 0.01, ^***^*P* < 0.001 compared with corresponding control; n.s. represents no statistical difference.

**Supplementary Figure S4**


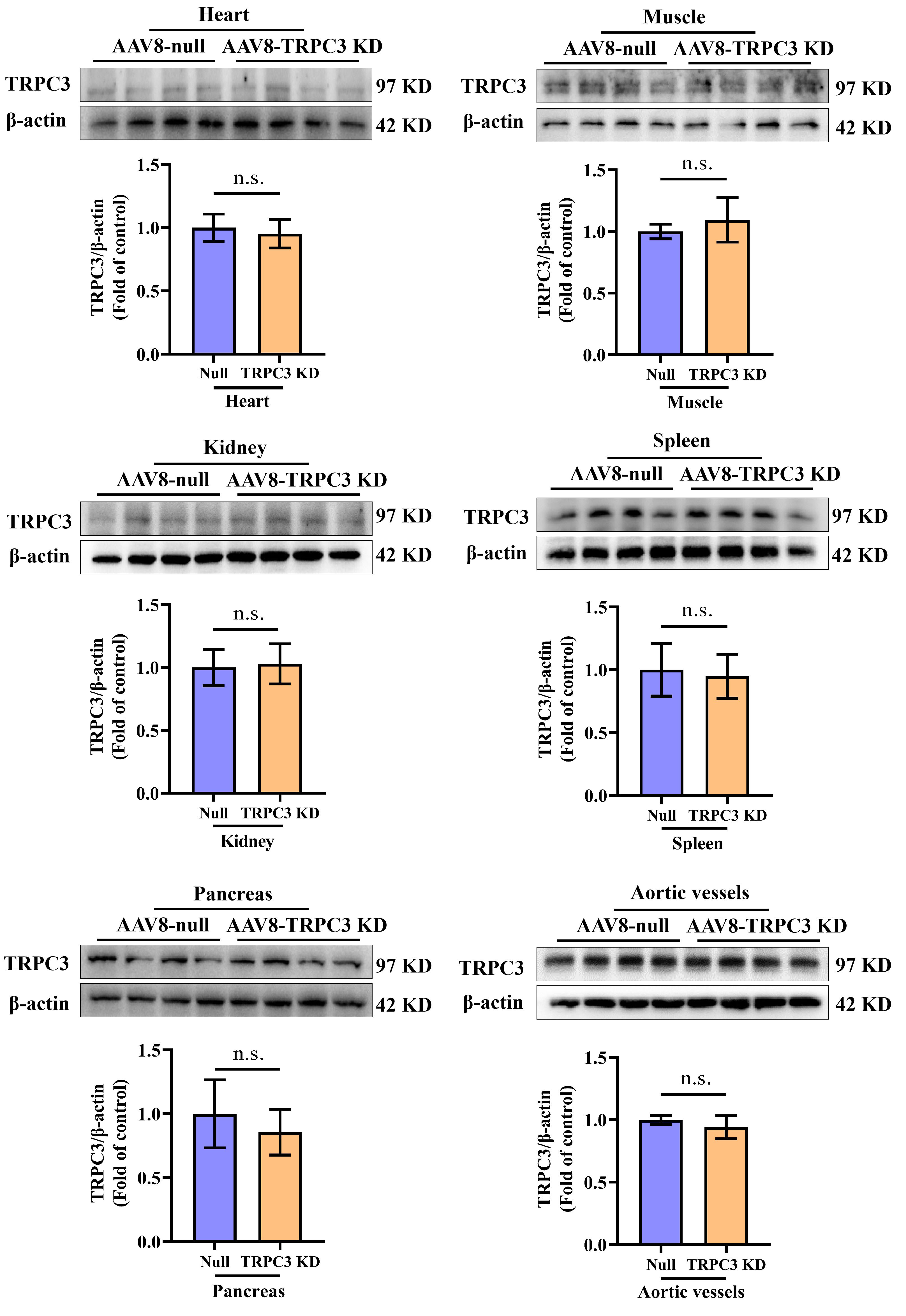


**Supplementary Figure S4** TRPC3 protein expression was detected in different tissues of liver-specific TRPC3 knockdown mice. Total lysates from different tissues were subjected to western blotting assay for TRPC3. Protein band intensity was quantified by ImageJ. Data are presented as means ± SD (*n* = 4). n.s. represents no statistical difference.

**Supplementary Figure S5**

**
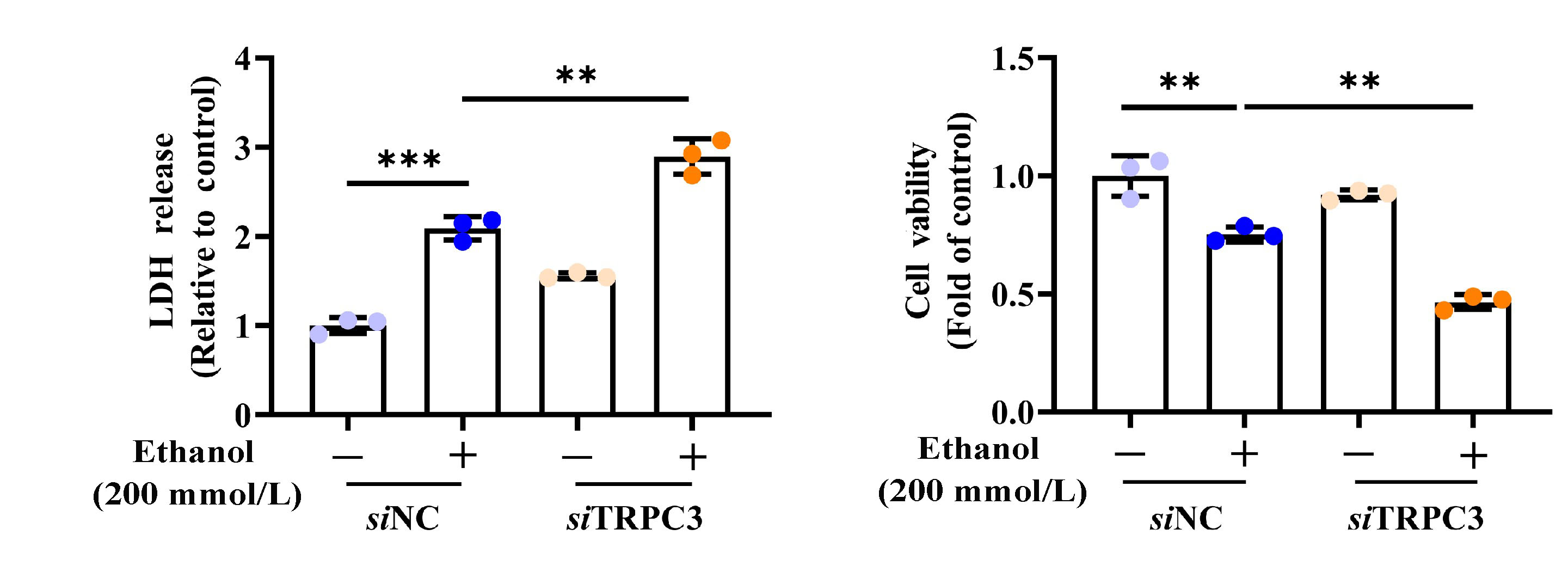
**

**Supplementary Figure S5** TRPC3 knockdown aggravates alcohol-induced cell damage. AML-12 cells were transfected with scramble siRNA (*si*NC) or TRPC3 siRNA (*si*TRPC3) for 48 h. Lactate dehydrogenase (LDH) release and cell viability were measured. Data are presented as means ± SD (*n* = 3). ^**^*p* < 0.01, ^***^*p* < 0.001 compared with corresponding control.

**Supplementary Figure S6**

**
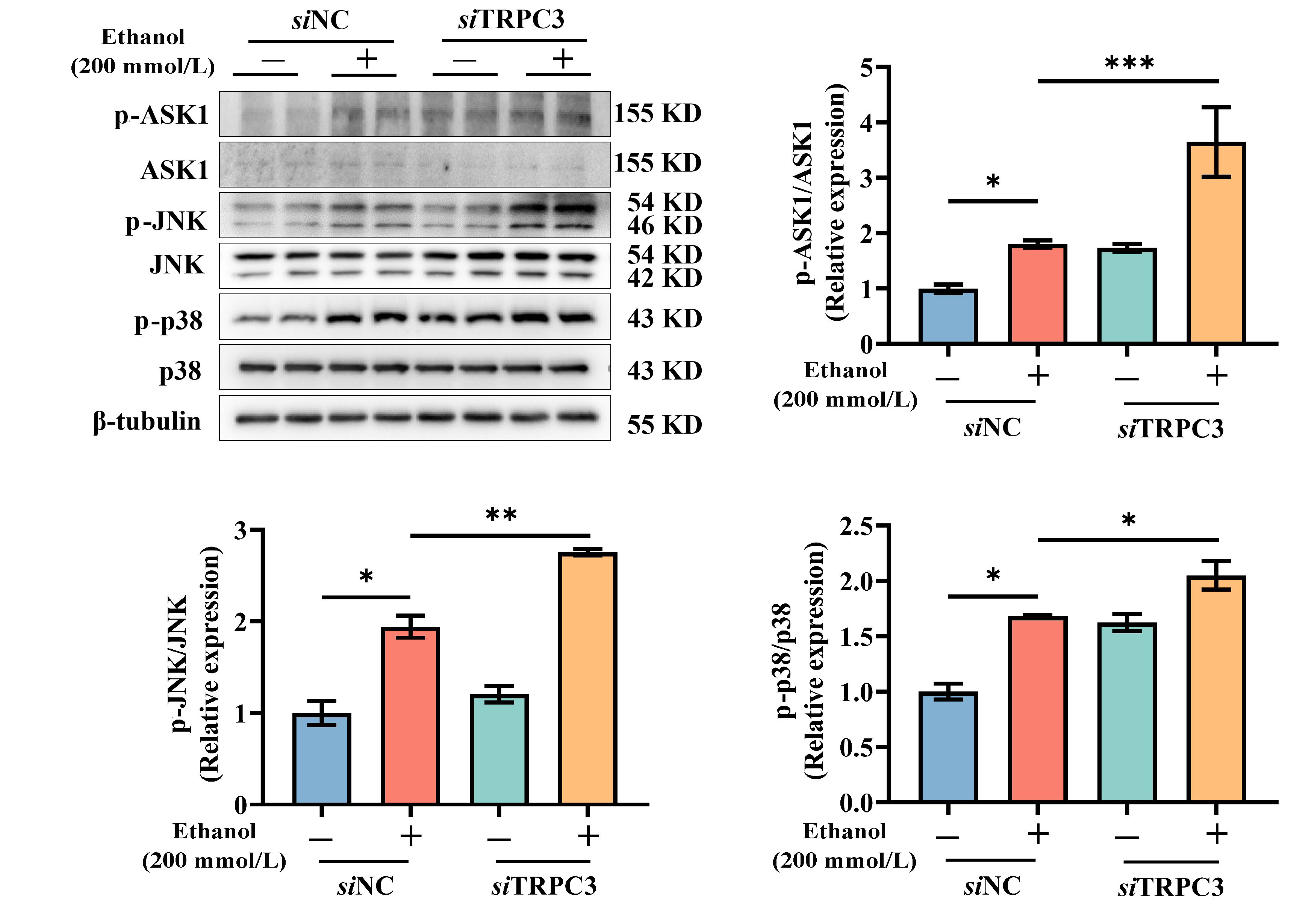
**

**Supplementary Figure S6** TRPC3 knockdown aggravates alcohol-induced hepatocyte apoptosis. AML-12 cells were treated with ethanol (200 mmol/L) for 48 h with NC *si*RNA or TRPC3 *si*RNA. Western blot was employed to detect the expression of phosphorylated-apoptosis signal-regulating kinase 1 (p-ASK1), p-JNK, and p-p38. Protein band intensity was quantified by ImageJ. Data are presented as means ± SD (*n* = 4). ^*^*P* < 0.05 and ^**^*P* < 0.01 compared with corresponding control.

**Supplementary Figure S7**

**
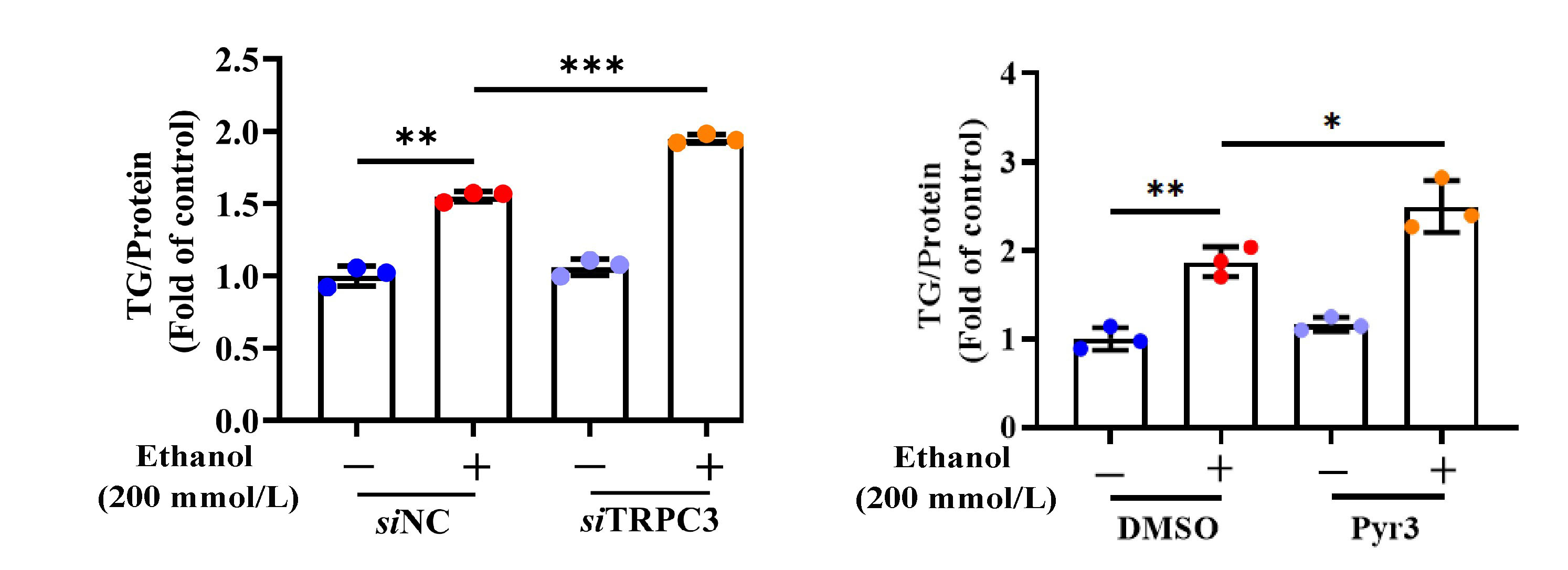
**

**Supplementary Figure S7** TRPC3 inhibition enhances ethanol-induced hepatic steatosis. (a) AML-12 cells were transfected with scramble siRNA (*si*NC) or TRPC3 siRNA (*si*TRPC3) for 48 h, and then cells were treated with ethanol (200 mmol/L) for 48 h. (b) AML-12 hepatocytes were treated with ethanol (200 mmol/L) for 48 h with or without a 2 h pretreatment of Pyr3 (TRPC3 antagonist, 10 μmol/L). Intracellular triglyceride (TG) was measured using a [commercial](file:///C:\Users\lisongtao\AppData\Local\Programs\baidu-translate-client\resources\app.asar\app.html) TG test kit. Data are presented as means ± SD (*n* = 3). ^*^*P* < 0.05, ^**^*P* < 0.01, ^***^*P* < 0.001 compared with corresponding control.

**Supplementary Figure S8**

**
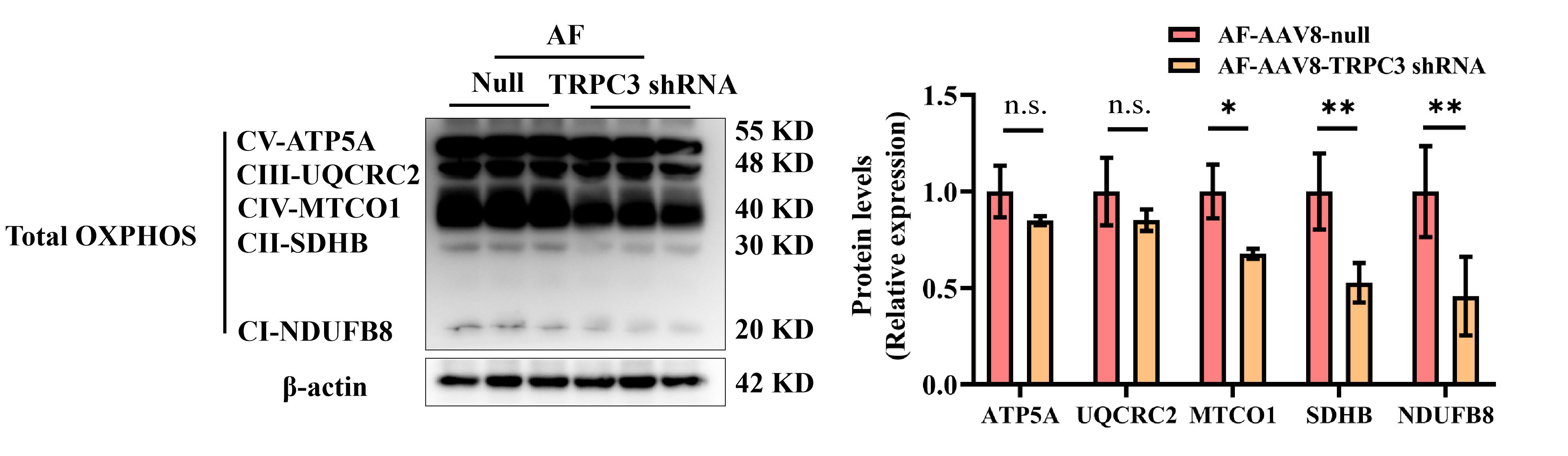
**

**Supplementary Figure S8** Loss of TRPC3 impairs the function of mitochondrial oxidative phosphorylation (OXPHOS). Western blot was performed to detect the expression of total OXPHOS (CI-NDUFB8, CII-SDHB, CIII-UQCRC2, CIV-MTCO1, and CV-ATP5A) in the liver of liver-specific TRPC3 knockdown mice in a chronic AF mouse model. Protein band intensity was quantified by ImageJ. Data are presented as means ± SD (*n* = 3). ^*^*P* < 0.05, ^**^*P* < 0.01 compared with corresponding control. n.s. represents no statistical difference.

**Supplementary Figure S9**

**
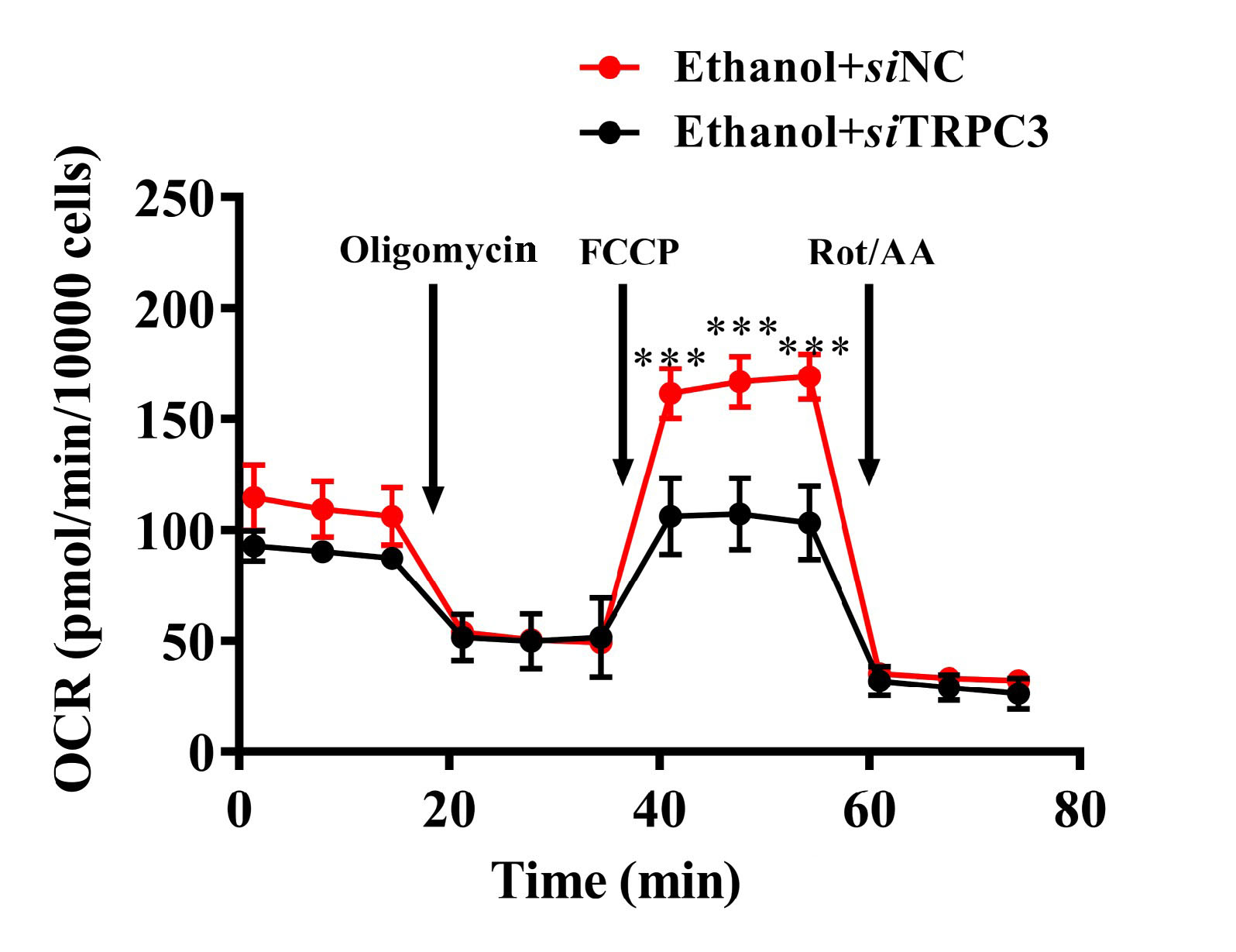
**

**Supplementary Figure S9** Loss of TRPC3 decreases OCR in AML-12 cells. AML-12 cells were transfected with scramble siRNA (*si*NC) or TRPC3 siRNA (*si*TRPC3) for 48 h, followed by treatment with ethanol (200 mmol/L) for 48 h. OCR value was measured with an XF96 Extracellular Flux Analyzer and normalized by cell number. ^***^*P* < 0.001 compared with corresponding control.

**Supplementary Figure S10**

**
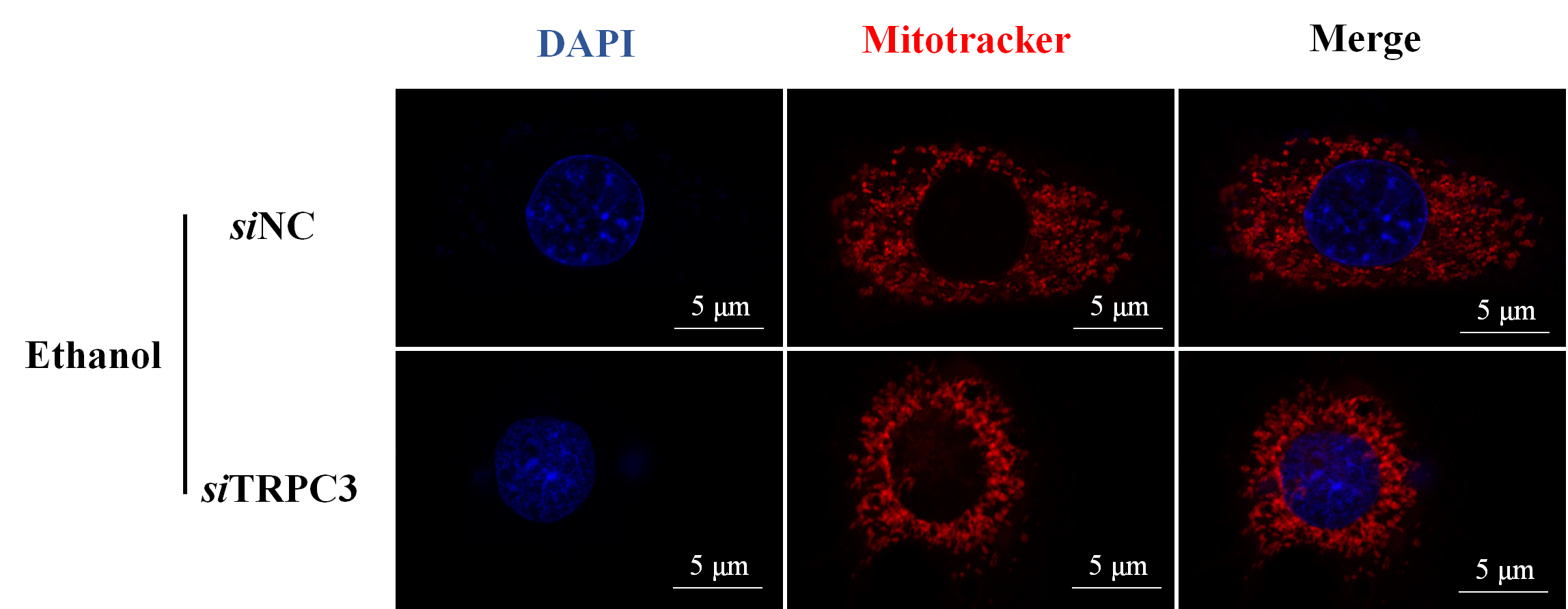
**

**Supplementary Figure S10** Loss of TRPC3 increases mitochondrial polymerization. AML-12 cells were transfected with scramble siRNA (*si*NC) or TRPC3 siRNA (*si*TRPC3) for 48 h, followed by treatment with ethanol (200 mM) for 48 h. Mitochondrial morphology was observed using MitoTracker staining. Images were captured with a laser confocal microscope.

**Supplementary Figure S11**

**
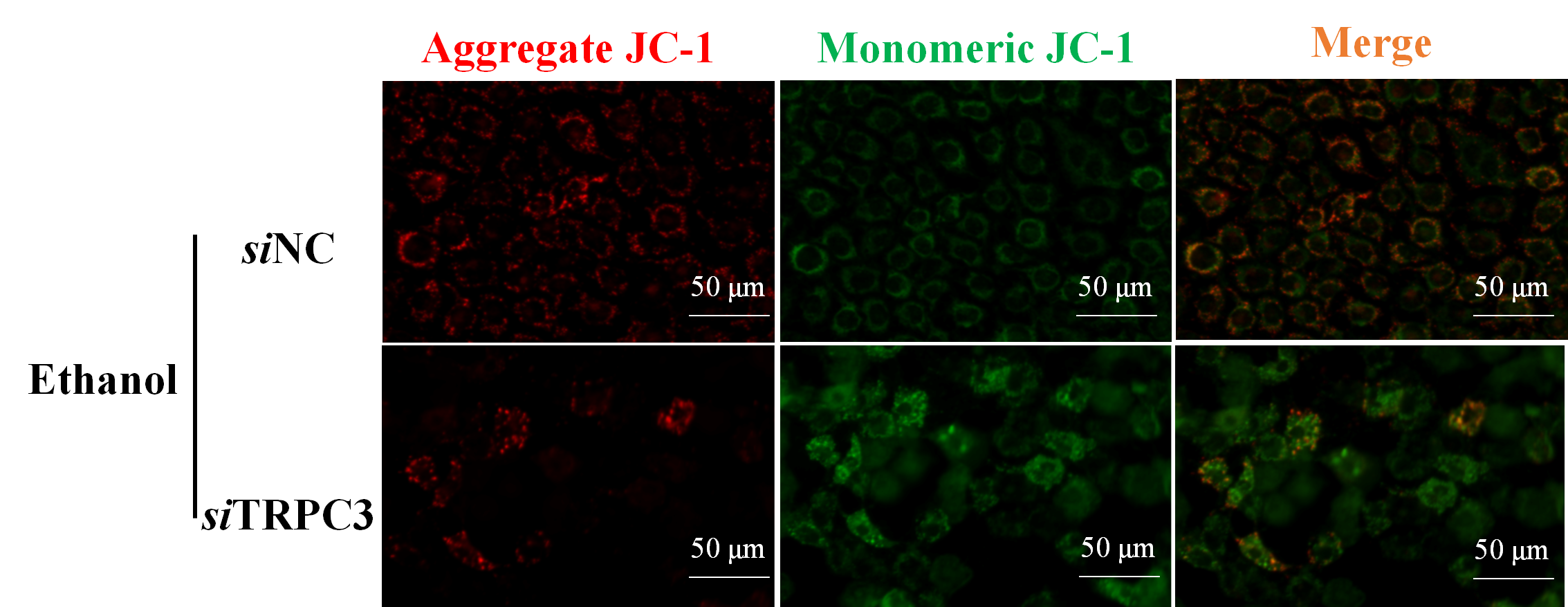
**
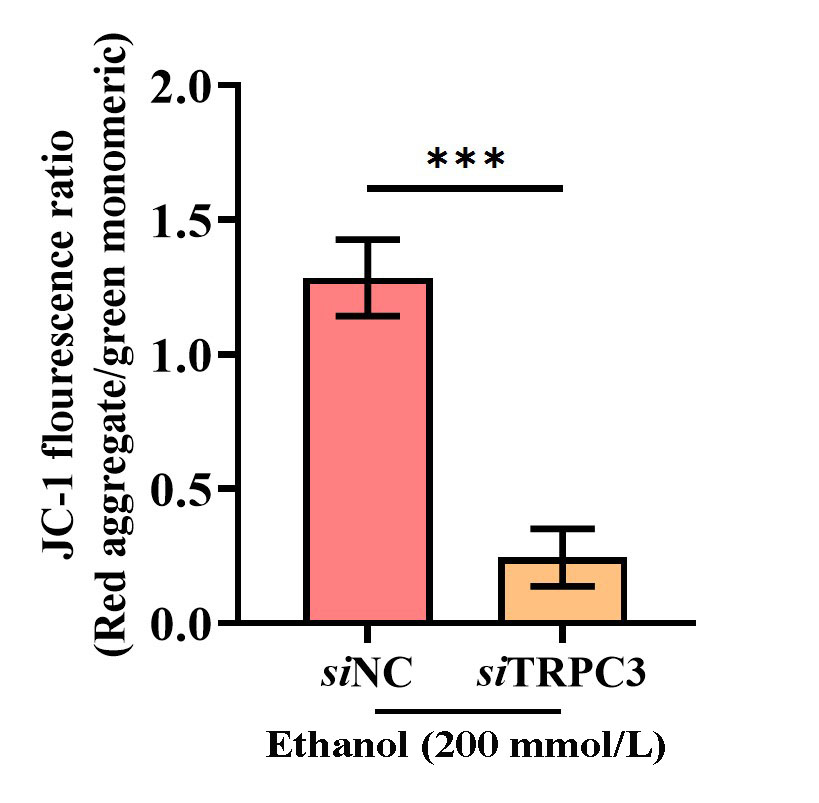


**Supplementary Figure S11** The loss of TRPC3 reduces mitochondrial membrane potential (MMP). AML-12 cells were transfected with scramble siRNA (*si*NC) or TRPC3 siRNA (*si*TRPC3) for 48 h, followed by treatment with ethanol (200 mmol/L) for 48 h. MMP was measured by JC-1 fluorescent probe. Images were obtained by using fluorescence microscopy acquisition. Cell fluorescence intensity was analyzed by ImageJ. Data are presented as means ± SD (*n* = 3). ^**^*P* < 0.01 compared with corresponding control. n.s. represents no statistical difference.

**Supplementary Figure S12**

**
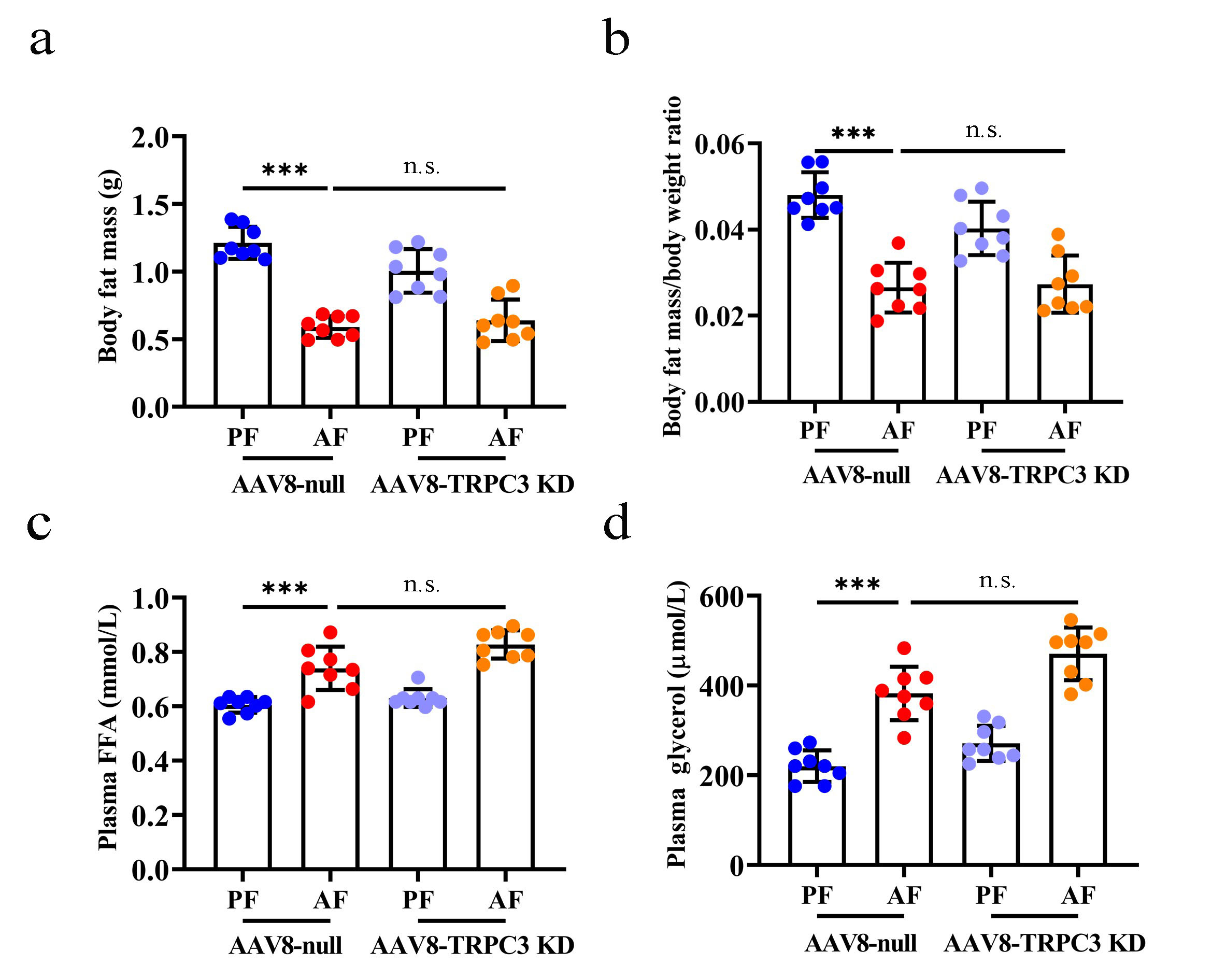
**

**Supplementary Figure S12** TRPC3 knockdown does not affect the lipolysis of adipose tissue in mice in challenge to alcohol. (a) Body fat mass (total weight of epididymal fat, subcutaneous fat, and brown adipose tissue). (b) Body fat mass/body weight ratio. (c) Plasma free fatty acid (FFA) level. (d) Plasma glycerol level. Data are presented as means ± SD (*n* = 8). ^*^*p* < 0.05, ^**^*p* < 0.01, ^***^*p* < 0.001 compared with corresponding control. n.s. represents no statistical difference.

**Supplementary Figure S13**

**
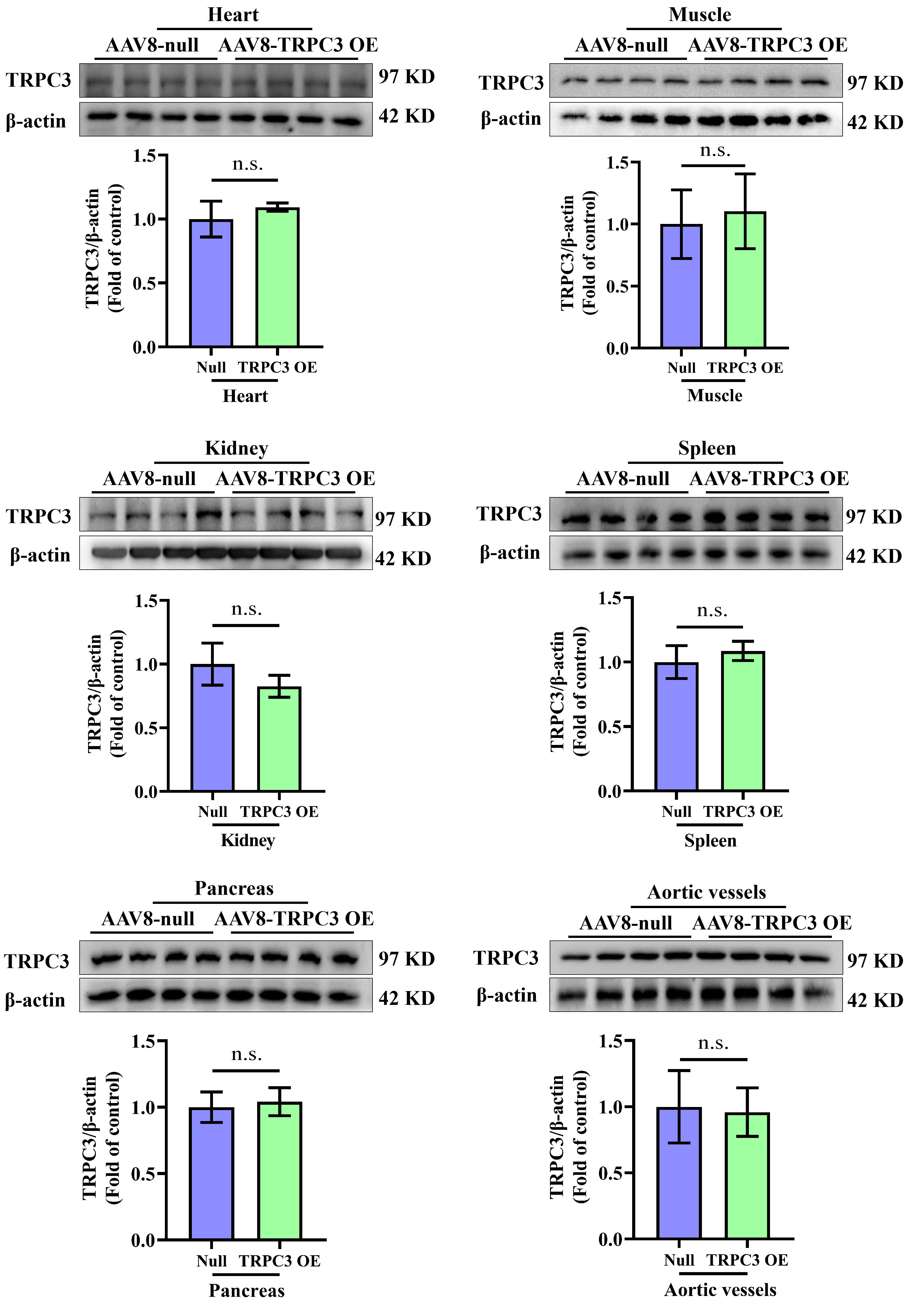
**

**Supplementary Figure S13** TRPC3 protein expression was detected in different tissues of liver-specific TRPC3 overexpression mice. Total lysates from different tissues were subjected to western blotting assay for TRPC3. Protein band intensity was quantified by ImageJ. Data are presented as means ± SD (*n* = 4). n.s. represents no statistical difference.

**Supplementary Figure S14**

**
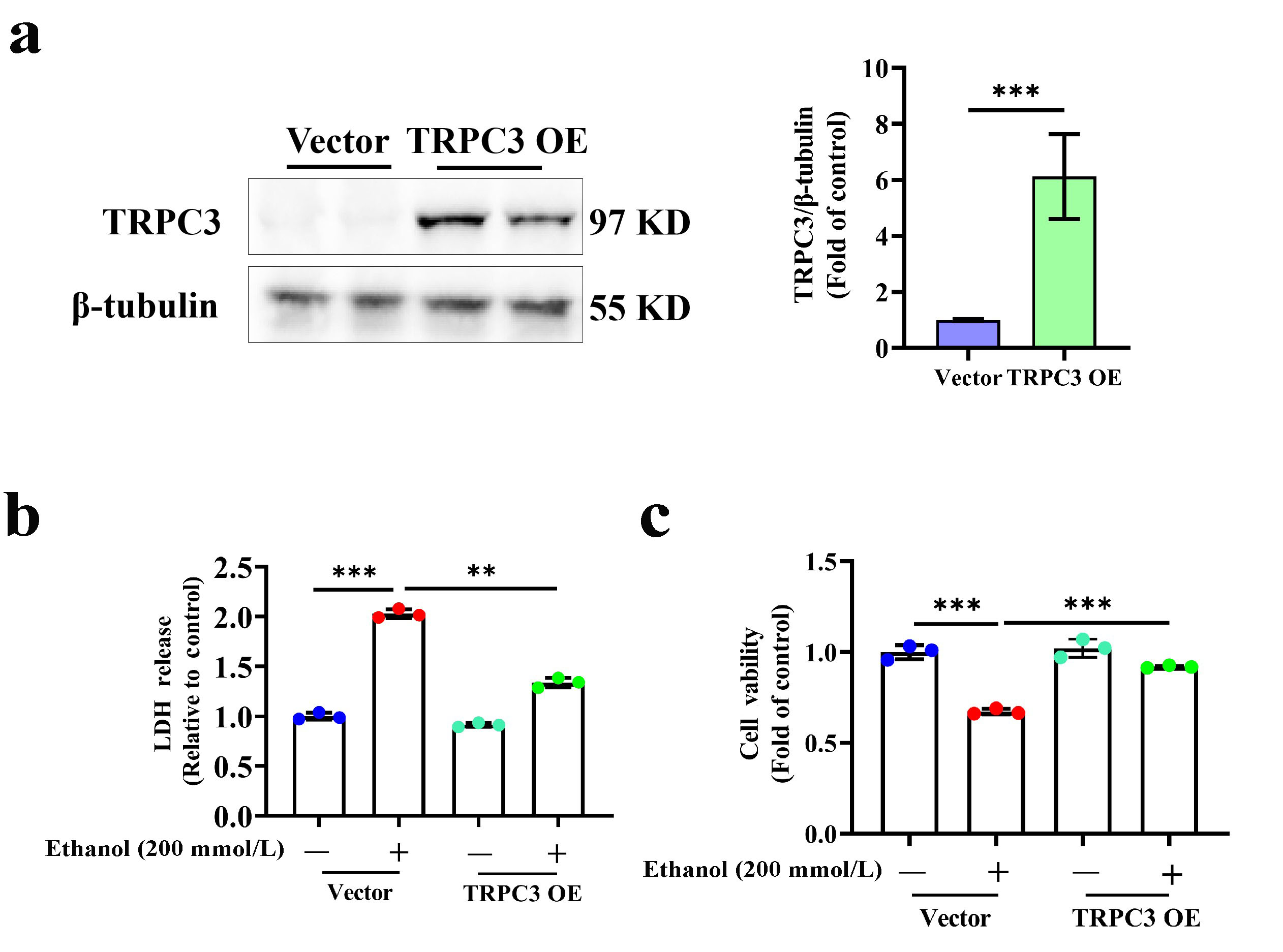
**

**Supplementary Figure S14** TRPC3 overexpression attenuates alcohol-induced cell damage. (a) AML-12 hepatocytes were transfected with lentiviral-constructed TRPC3 overexpression plasmid for 48 h. TRPC3 overexpression efficiency was detected in ALM-12 hepatocytes. Protein band intensity was quantified by ImageJ. (b and c) AML12 cells were transfected with TRPC3 overexpressed lentiviruses for 48 h, before being exposed to ethanol, and LDH release and cell viability were measured. Data are presented as means ± SD (*n* = 3). ^**^*P* < 0.01, ^***^*P* < 0.001 compared with corresponding control;

**Supplementary Figure S15**

**
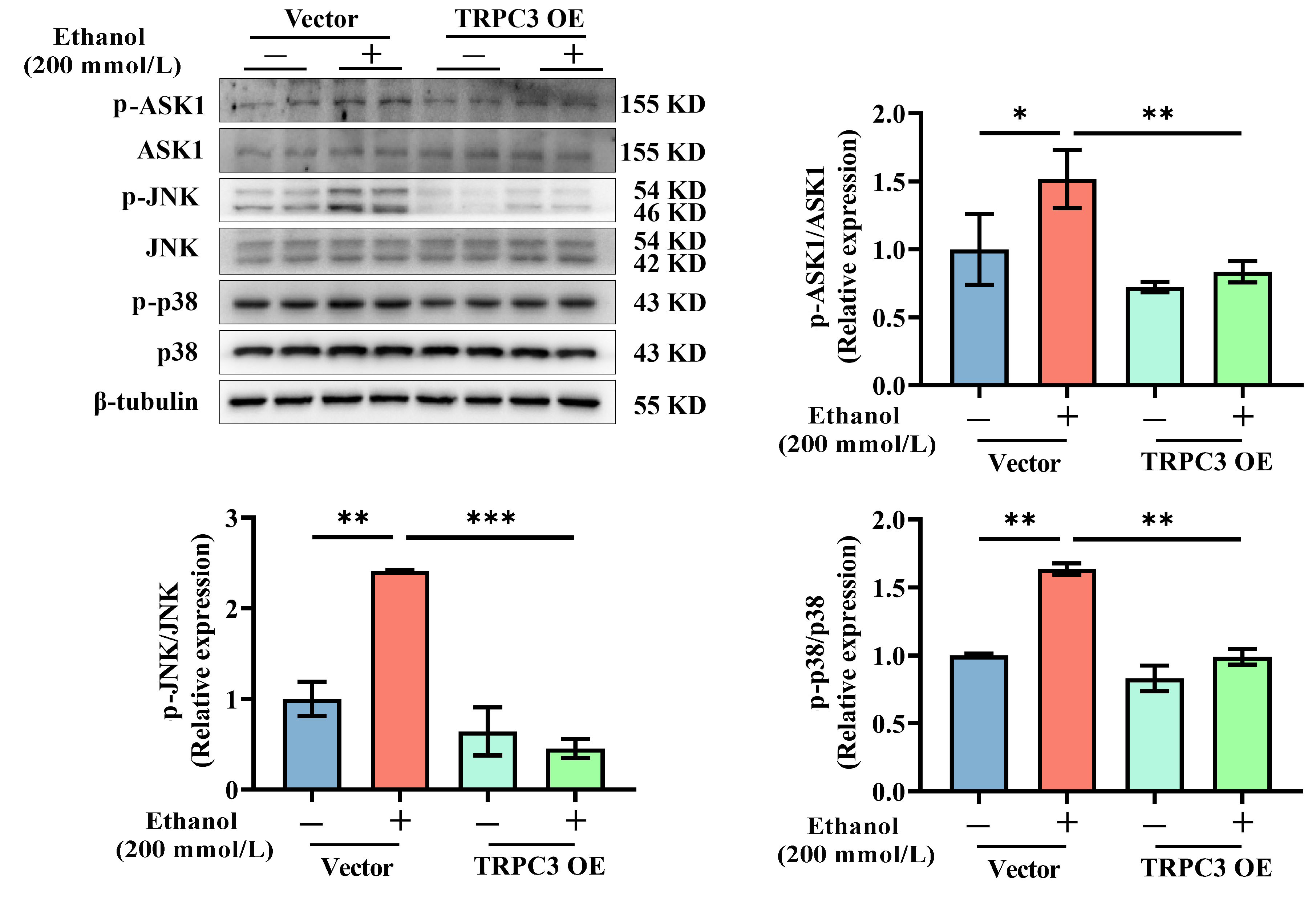
**

**Supplementary Figure S15** TRPC3 overexpression improves alcohol-induced hepatocyte apoptosis. AML-12 hepatocytes were transfected with lentiviral-constructed TRPC3 overexpression plasmid, and ethanol intervention was performed 48 h after transfection. Western blot was employed to detect the expression of p-ASK1, p-JNK, and p-p38. Protein band intensity was quantified by ImageJ. Data are presented as means ± SD (*n* = 4). ^*^*P* < 0.05, ^**^*P* < 0.01, ^***^*P* < 0.001 compared with corresponding control.

**Supplementary Figure S16**

**
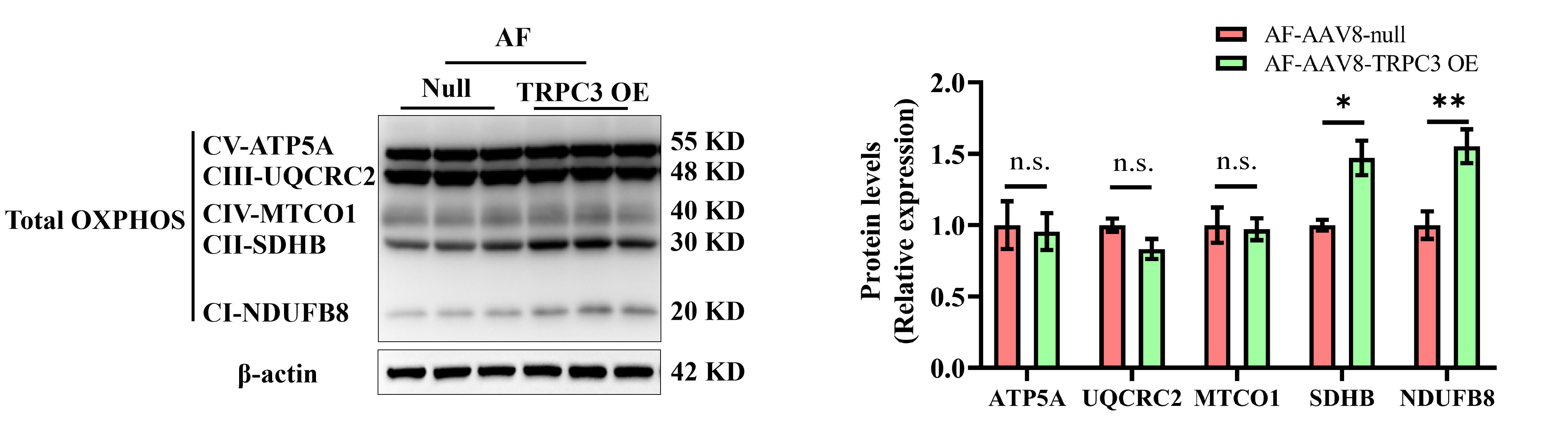
**

**Supplementary Figure S16** TRPC3 overexpression restores mitochondrial OXPHOS. AML-12 hepatocytes were transfected with lentiviral-constructed TRPC3 overexpression plasmid for 48 h, followed by treatment with ethanol (200 mmol/L) for 48 h. Western blot was performed to detect the expression of total OXPHOS (CI-NDUFB8, CII-SDHB, CIII-UQCRC2, CIV-MTCO1, and CV-ATP5A). Protein band intensity was quantified by ImageJ. Data are presented as means ± SD (*n* = 3). ^*^*P* < 0.05, ^**^*P* < 0.01 compared with corresponding control. n.s. represents no statistical difference.

**Supplementary Figure S17**

**
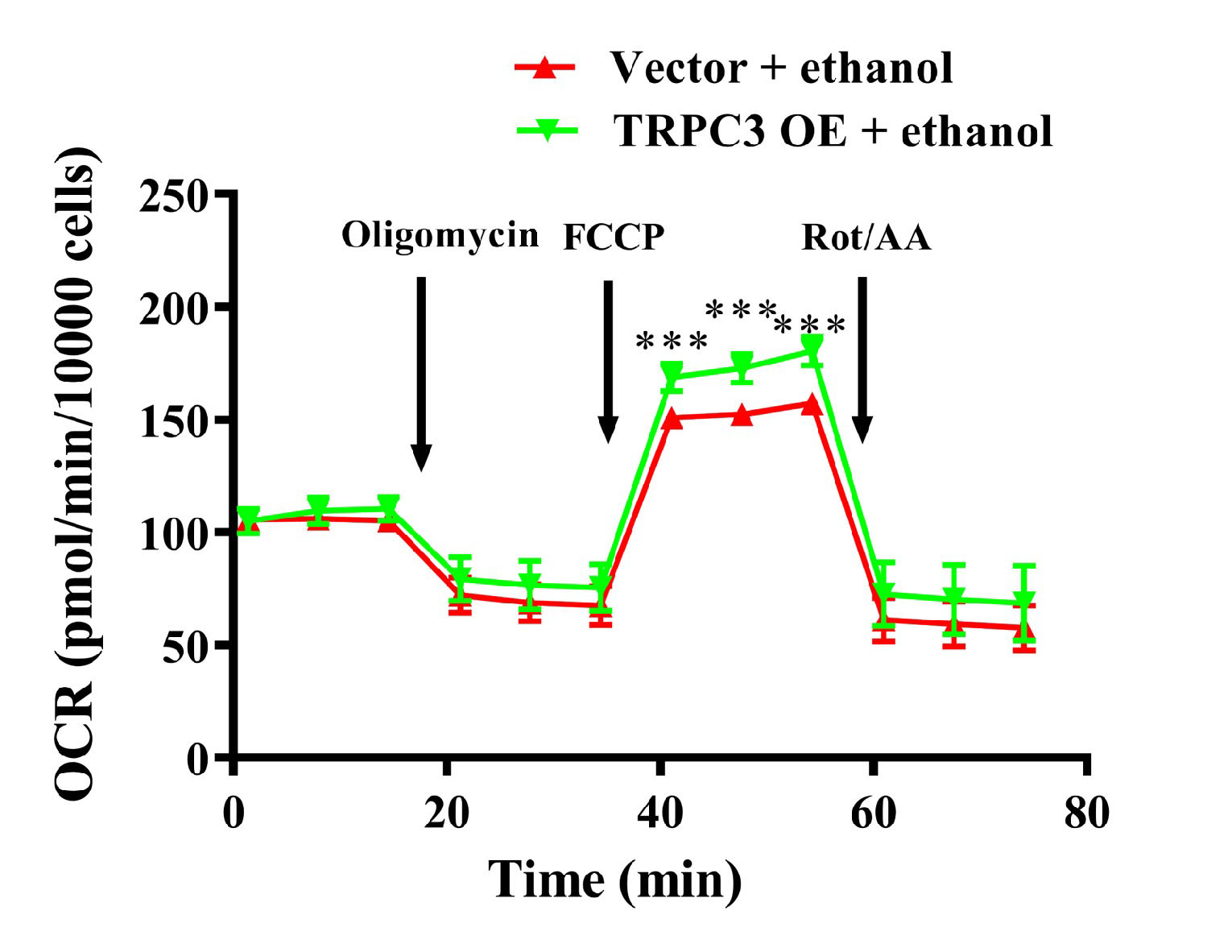
**

**Supplementary Figure S17** Overexpression of TRPC3 reduces ethanol-increased oxygen consumption in AML-12 cells. AML-12 hepatocytes were transfected with lentiviral-constructed TRPC3 overexpression plasmid for 48 h, followed by treatment with ethanol (200 mmol/L) for 48 h. OCR value was measured with an XF96 Extracellular Flux Analyzer and normalized by cell number. ^***^*P* < 0.001 compared with corresponding control.

**Supplementary Figure S18**

**
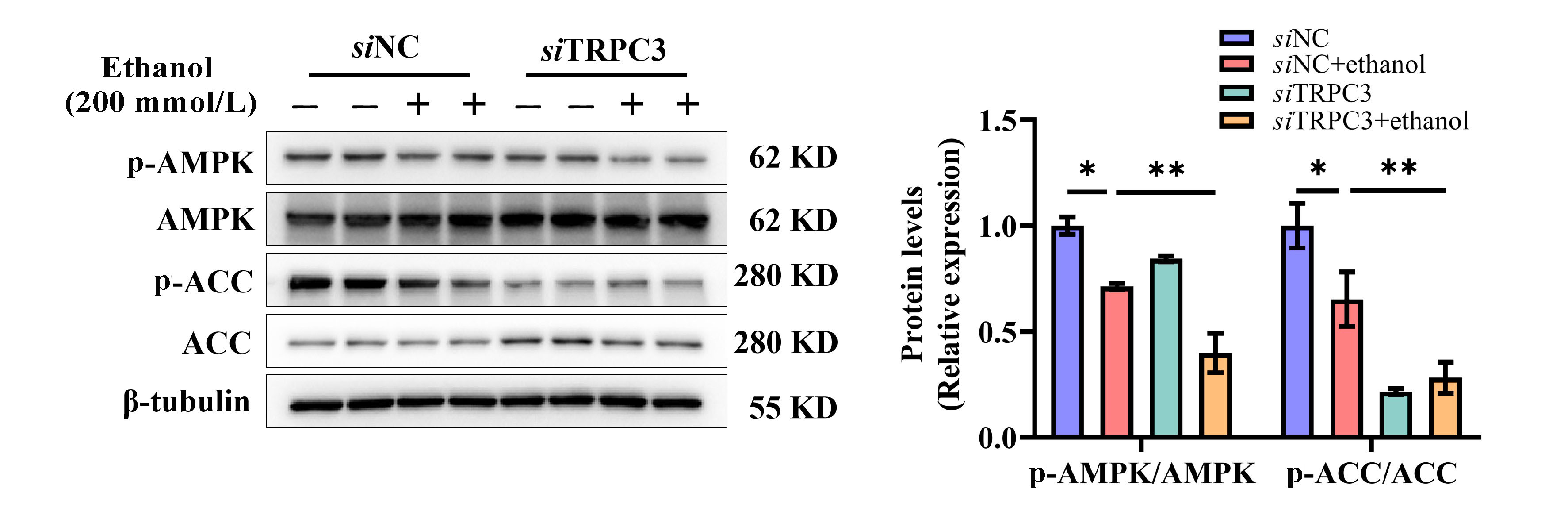
**

**Supplementary Figure S18** TRPC3 knockdown aggravates ethanol-decreased phosphorylation of AMPK and 1-aminocyclopropane-1-carboxylate (ACC). AML-12 cells were transfected with scramble siRNA (*si*NC) or TRPC3 siRNA (*si*TRPC3) for 48 h, followed by treatment with ethanol (200 mmol/L) for 48 h. Western blot was employed to detect the expression of p-AMPK and p-ACC. Protein band intensity was quantified by ImageJ. Data are presented as means ± SD (*n* = 4). ^*^*P* < 0.05, ^**^*P* < 0.01 compared with corresponding control.

**Supplementary Figure S19**

**
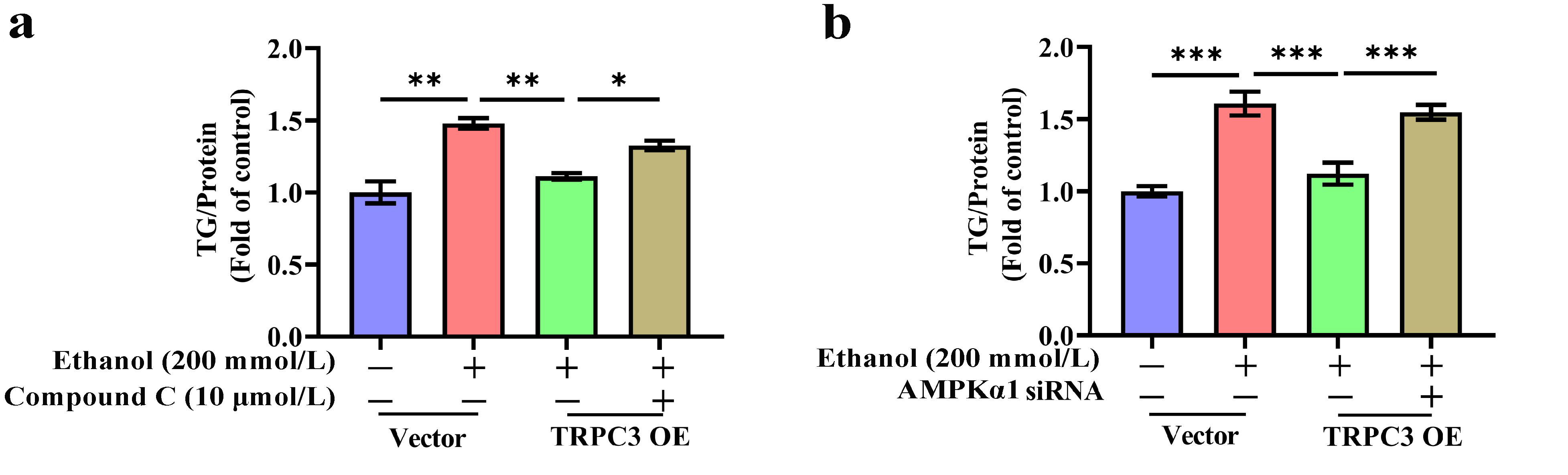
**

**Supplementary Figure S19** Inhibition of AMPK blockes TRPC3 overexpression to protect against alcohol-induced lipid deposition. AML-12 cells were exposed to ethanol with TRPC3 overexpression by lentiviruses transfection. Compound C (10 μmol/L) was added 2 h before ethanol treatment or transfected AMPKα *si*RNA for 48 h before ethanol treatment. Intracellular TG was detected. ^*^*P* < 0.05, ^**^*P* < 0.01, ^***^*P* < 0.001 compared with corresponding control.

**Supplementary Figure S20**

**
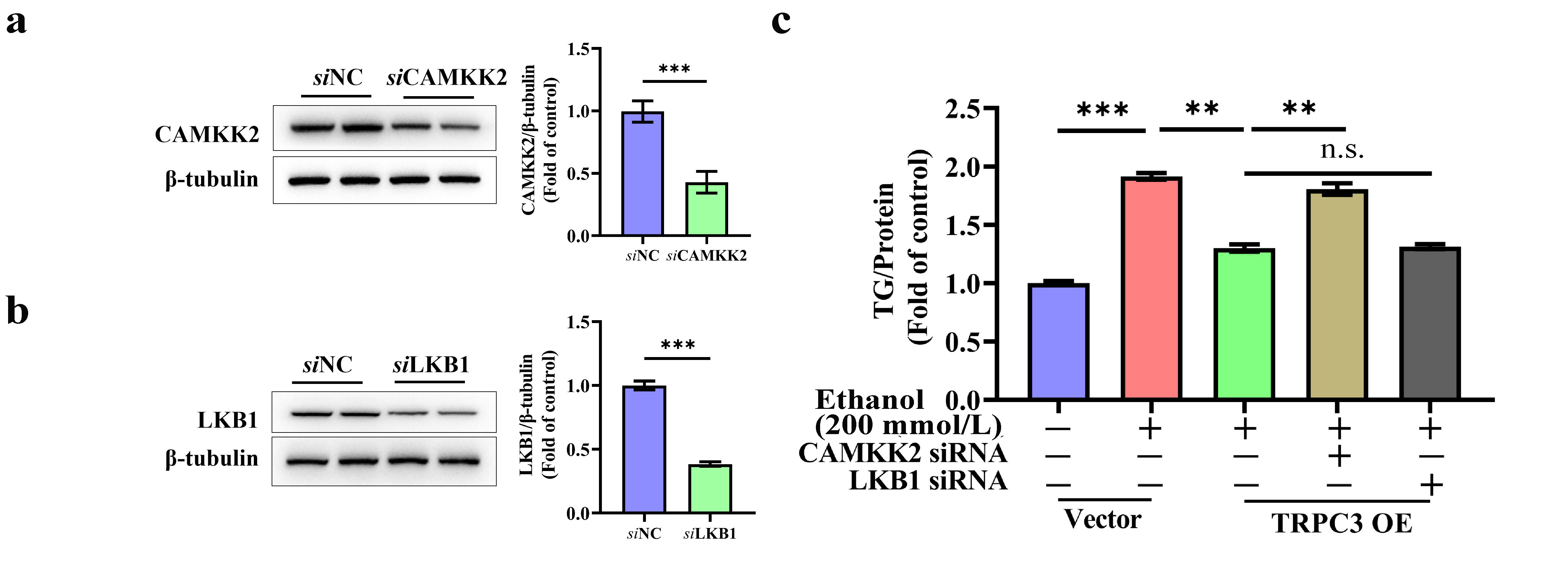
**

**Supplementary Figure S20** Inhibition of CAMKK2 blockes TRPC3 overexpression to protect against alcohol-induced lipid deposition. AML-12 cells were transfected with scramble siRNA (*si*NC), CAMKK2 siRNA (*si*CAMKK2), or LKB1 siRNA (*si*LKB1) for 48 h, followed by treatment with ethanol (200 mmol/L) for 48 h. (a and b) CAMKK2 and LKB1 knockdown efficiencies were detected in ALM-12 hepatocytes. Western blot was performed to analyze the expression of CAMKK2 and LKB1. (C) Intracellular TG was detected. ^**^*p* < 0.01, ^***^*p* < 0.001 compared with corresponding control. n.s. represents no statistical difference.

**Supplementary Figure S21**

**
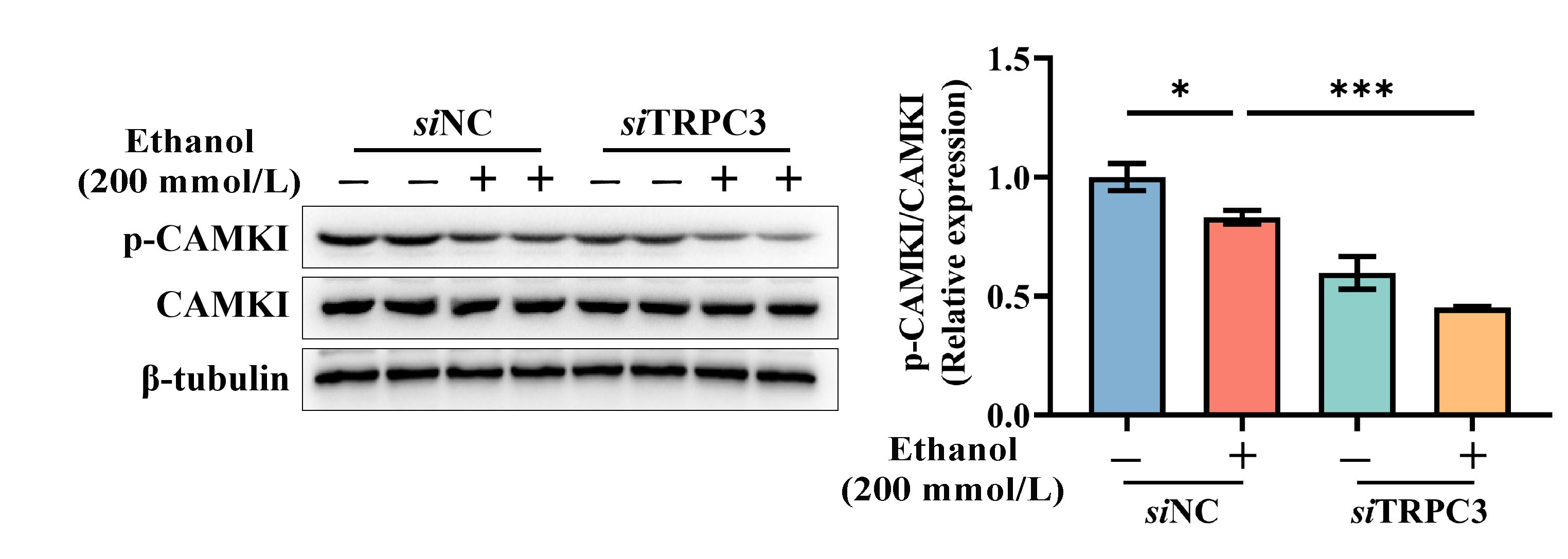
**

**Supplementary Figure S21** TRPC3 knockdown exacerbates ethanol-decreased calmodulin-dependent protein kinase I (CAMKI) phosphorylation. AML-12 cells were transfected with scramble siRNA (*si*NC) or TRPC3 siRNA (*si*TRPC3) for 48 h, followed by treatment with ethanol (200 mmol/L) for 48 h. Western blot was performed to detect the expression of p-CAMKI. Protein band intensity was quantified by ImageJ. Data are presented as means ± SD (*n* = 4). ^*^*P* < 0.05, ^***^*P* < 0.001 compared with corresponding control.

**Supplementary Figure S22**

**
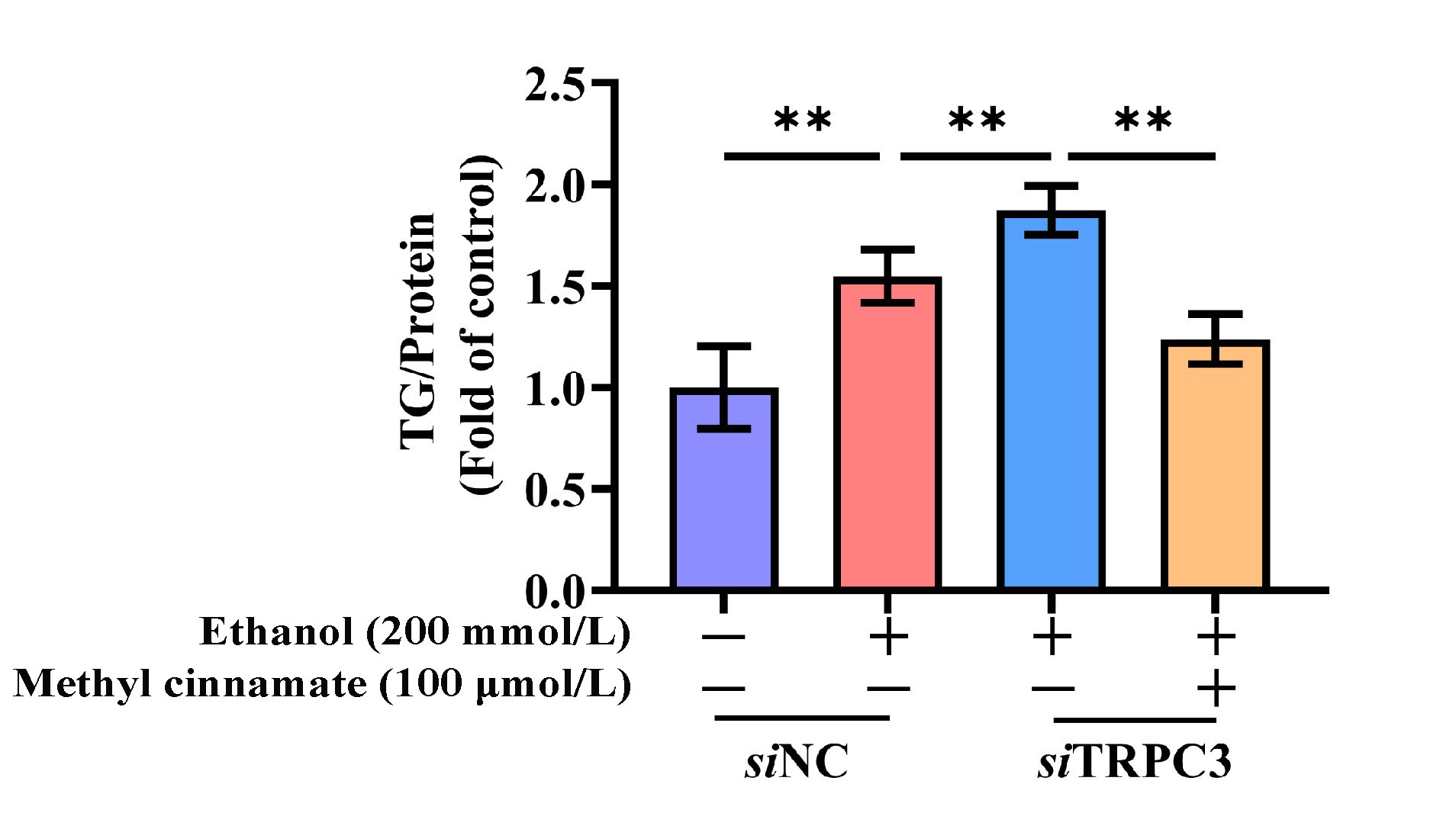
**

**Supplementary Figure S22** Activation of CAMKK2 ameliorates TRPC3 knockdown aggravated alcohol-induced lipid deposition. AML-12 cells were transfected with scramble siRNA (*si*NC) or TRPC3 siRNA (*si*TRPC3) for 48 h, followed by treatment with ethanol (200 mmol/L) for 48 h. CAMKK2 agonist (methyl cinnamate, 100 μmol/L) was added 2 h before ethanol treatment. Intracellular TG was detected. ^**^*P* < 0.01 compared with corresponding control.

**Supplementary Figure S23**

**
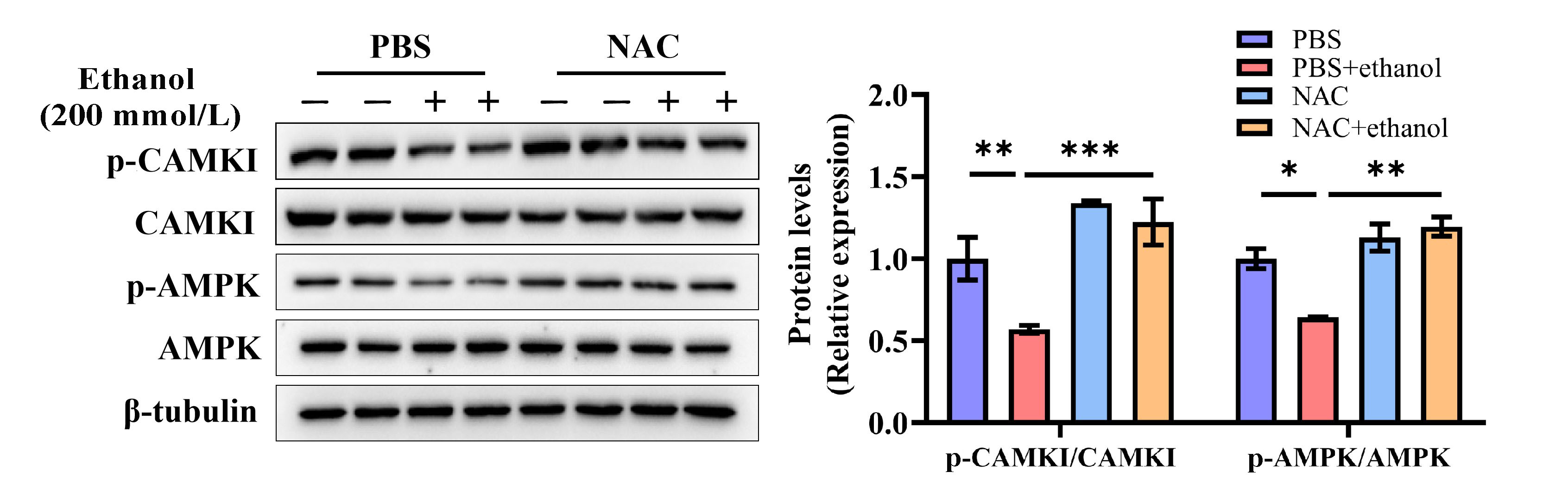
**

**Supplementary Figure S23** NAC supplementation reverses phosphorylation levels of CAMKI and AMPK in ethanol-treated AML-12 cells. AML12 cells were treated with ethanol (200 mmol/L) for 48 h with or without a 2-h pretreatment with NAC (5 mmol/L). Western blot was employed to detect the expression of p-CAMKI and p-AMPK. Protein band intensity was quantified by ImageJ. Data are presented as means ± SD (*n* = 4). ^*^*P* < 0.05, ^**^*P* < 0.01, ^***^*P* < 0.001 compared with corresponding control.

**Supplementary Figure S24**

**
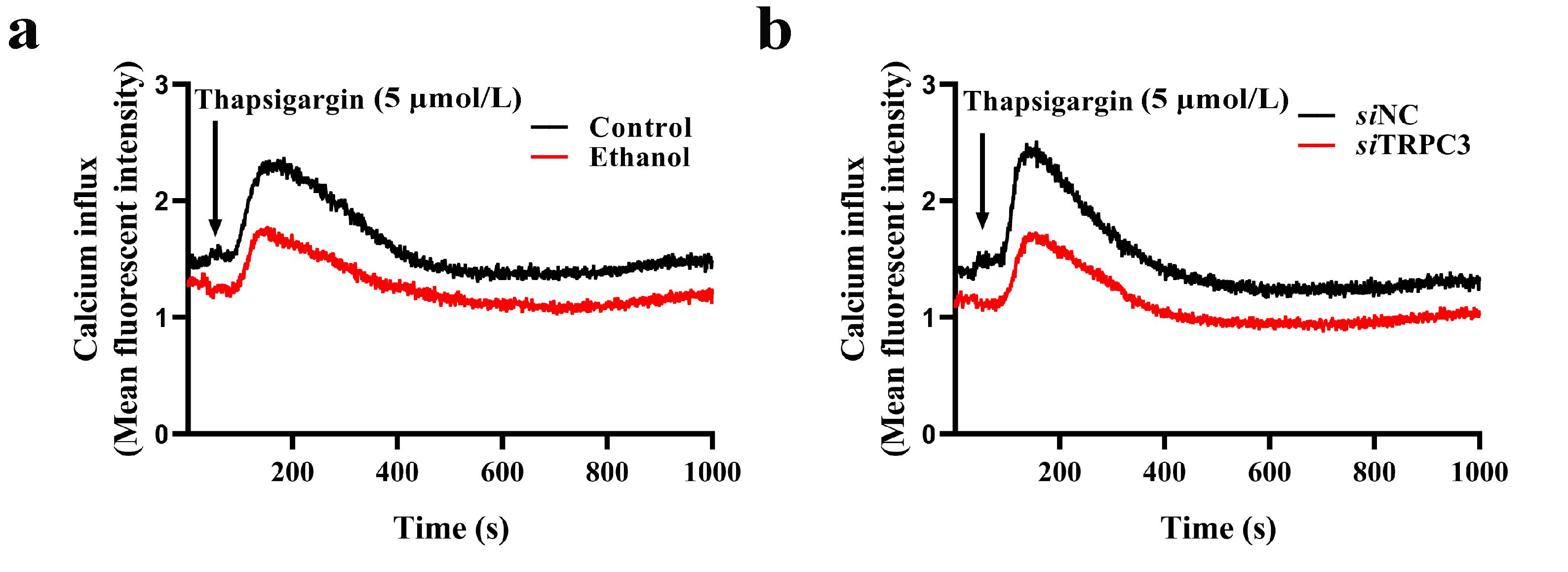
**

**Supplementary Figure S24** Ethanol intervention or silencing of TRPC3 inhibits thapsigargin-induced calcium inward flow. (a) Ethanol treatment of AML-12 cells for 48 h. (b) AML-12 cells were transfected with scramble siRNA (*si*NC) or TRPC3 siRNA (*si*TRPC3) for 48 h. Intracellular calcium response of AML-12 cells with alcohol-exposed or silencing TRPC3 was recorded by Fluo4 fluorescence in response to Fluo4/AM. Cells were treated with thapsigargin (5 μmol/L) at the 60-s time point (indicated by arrow). Data are presented as means ± SD (*n* = 3).

**Supplementary Tables**

**Supplementary Table S1** Primers designed for Real-Time PCR (mouse).

| Gene name | | Forward (5’-3’) | Reverse (5’-3’) |
| --- | --- | --- | --- |
| *18S* | AGGTCTGTGATGCCCTTAGA | | GAATGGGGTTCAACGGGTTA |
| *Trpc1* | ACGGTTGTCAGAAACTTATGGA | | GCTTGGGCAAAGACACATCC |
| *Trpc3* | CTAGGCGGTCATGATTCCCA | | GGAGGCCAACAGGTGACATA |
| *Trpc4* | AAGTGACGAGATGTCGAAGGA | | GTCTTCAATGAGAGTCGCTGTTT |
| *Trpc5* | GTGTATCCAGTTCGGAGGTAGA | | CCTCGCTTGATAAGGCAATGA |
| *Trpc6* | GGCGGCTCTCTAAAGGCTG | | TGGGGTAGTAGCCATACGGTG |
| *Trpc7* | TCCTGGACTCGGCTGAGTATG | | GCAGTTGAAATTGAGGGTCTTGG |
| *Srebp1c* | CCACCTCGTGAACGAAGATT | | ACCACCACGACCTCGAATAG |
| *Fasn* | GGAGGTGGTGATAGCCGGTAT | | TGGGTAATCCATAGAGCCCAG |
| *Scd1* | TTCTTGCGATACACTCTGGTGC | | CGGGATTGAATGTTCTTGTCGT |
| *Dgat1* | GTGCCATCGTCTGCAAGATTC | | GCATCACCACACACCAATTCAG |
| *Dgat2* | AGTGGCAATGCTATCATCATCGT | | TCTTCTGGACCCATCGGCCCCAGGA |
| *Fatp2* | TCCTCCAAGATGTGCGGTACT | | TAGGTGAGCGTCTCGTCTCG |
| *Fatp5* | CTACGCTGGCTGCATATAGATG | | CCACAAAGGTCTCTGGAGGAT |
| *Cpt1α* | CTATGCGCTACTCGCTGAAGG | | GGCTTTCGACCCGAGAAGA |
| *Pparα* | TGCCTTCCCTGTGAACTGAC | | TGGGGAGAGAGGACAGATGG |
| *Vldlr* | GGAGATGCGATGGTGAAAAT | | AGAGGTGCTGCACTGGAACT |
| *Atgl* | CAACGCCACTCACATCTACGG | | GGACACCTCAATAATGTTGGCAC |
| *Hsl* | TCCCTCAGTATCTAGGCCAGA | | GGCTCATTTGGGAGACTTTGTTT |
| *Il-1β* | GAAATGCCACCTTTTGACAGTG | | TGGATGCTCTCATCAGGACAG |
| *Il-6* | GATGCTACCAAACTGGATATAATC | | GGTCCTTAGCCACTCCTTCTGTG |
| *Tnf-α* | CCCTCACACTCAGATCATCTTC | | GTTGGTTGTCTTTGAGATCCAT |
| *Il-17a* | GCTCCAGAAGGCCCTCAGA | | CTCTCCACCGCAATGAAGACCCTGA |
| *Nos2* | GAGGCCCAGGAGGAGAGAGATCCG | | TCCATGCAGACAACCTTGGTGTTG |
| *Ccl2* | AGGTCCCTGTCATGCTTCTG | | TCTGGACCCATTCCTTCTTG |
| *Ccl5* | TGCCCACGTCAAGGAGTATTT | | TTCTCTGGGTTGGCACACACT |
| *Cxcl2* | GCTTGTCTCAACCCCGCATC | | TGGCCTCTGCAGCTGTGTGCT |
| *Cxcl8* | AGACAGCAGAGCACACAAGC | | ATGGTTCCTTCCGGTGGT |
| *Acta2* | CTGACAGAGGCACCACTGAA | | CATCTCCAGAGTCCAGCACA |
| *Tgf-β* | TGCTAATGGTGGACCGCAA | | CACTGCTTCCCGAATGTCTGA |
| *Col1a1* | TGTGTTCCCTACTCAGCCGTCT | | CATCGGTCATGCTCTCTCCAA |
| *Col2a1* | TGAGGTCTGGGTAAAGGCAA | | GTATGAGGTCACCGTCCAGG |
| *Col3a1* | TAGGACTGACCAAGGTGGCT | | GGAACCTGGTTTCTTCTCACC |
| *Col4a1* | CACATTTTCCACAGCCAGAG | | GTCTGGCTTCTGCTGCTCTT |
| *Col5a1* | CTTCGCCGCTACTCCTGTTC | | CCCTGAGGGCAAATTGTGAAAA |
| *U6* | CTCGCTTCGGCAGCACATATAC | | AATATGGAACGCTTCACGAATTTG |
| *miR-218-5p* | CGGTTGTGCTTGATCTAACCATGT | | |
| *miR-137-3p* | GCGCTTATTGCTTAAGAATACGCGT | | |
| *miR-339-5p* | ATTATCCCTGTCCTCCAGGAGCT | | |
| *miR-325-3p* | CGGCGTTTATTGAGCACCTCCTATCAA | | |
| *miR-3097-5p* | ATTACACAGGTGGGAAGTGTGTGTC | | |
| *miR-1264-3p* | CGGCAAATCTTATTTGAGCACCTGT | | |
| *miR-470-3p* | CGAACCAGTACCTTTCTGAGAAGA | | |
| *miR-380-3p* | GCGCTATGTAGTATGGTCCACATCTT | | |

**Supplementary Table S2** Antibodies list.

| Name | Citation | Supplier | Cat no. | Clone no. |
| --- | --- | --- | --- | --- |
| TRPC1 | WB | Santa Cruz Biotechnology | sc-133076 | E-6 |
| TRPC3 | WB | Santa Cruz Biotechnology | sc-514670 | C-5 |
| TRPC4 | WB | Proteintech Group | 21349-1-AP | Polyclonal |
| TRPC5 | WB | Santa Cruz Biotechnology | sc-293259 | 1C8 |
| TRPC6 | WB | Santa Cruz Biotechnology | sc-515837 | B-10 |
| TRPC7 | WB | Abclonal | A17738 | Polyclonal |
| P-ASK1 | WB | Abcam | ab39402 | S966 |
| ASK1 | WB | Cell signaling technology | 8662S | D11C9 |
| P-JNK | WB | Cell signaling technology | [9255](https://www.cellsignal.cn/products/primary-antibodies/phospho-sapk-jnk-thr183-tyr185-g9-mouse-mab/9255?site-search-type=Products&N=4294956287&Ntt=jnk&fromPage=plp)S | Thr183/Tyr185 |
| JNK | WB | Cell signaling technology | 9252S | Polyclonal |
| P-p38 | WB | Cell signaling technology | 4511S | Thr180/Tyr182 |
| p38 | WB | Cell signaling technology | 8690S | D13E1 |
| P-AMPKalpha | WB | Cell signaling technology | 2535S | Thr172 40H9 |
| AMPKalpha | WB | Cell signaling technology | 2532S | Polyclonal |
| P-ACC | WB | Cell signaling technology | 3661S | Ser79 |
| ACC | WB | Cell signaling technology | 3662S | Polyclonal |
| P-CAMKI | WB | Abcam | ab62215 | T177 |
| CAMKI | WB | Abcam | ab68234 | EPR2217Y |
| SREBP-1 | WB | Santa Cruz Biotechnology | sc-365513 | A-4 |
| DGAT2 | WB | Santa Cruz Biotechnology | sc-293211 | 4C1 |
| VLDLR | WB | Proteintech | 19493-1-AP | Polyclonal |
| ATGL | WB | Santa Cruz Biotechnology | sc-365278 | F-7 |
| OXPHOS (NDUFB8) | WB | Abcam | ab110413 | 20E9DH10C12 |
| OXPHOS (MTCO1) | WB | Abcam | ab110413 | 1D6E1A8 |
| OXPHOS (SDHB) | WB | Abcam | ab110413 | 21A11AE7 |
| OXPHOS (UQCRC2) | WB | Abcam | ab110413 | 13G12AF12BB11 |
| OXPHOS (ATP5A) | WB | Abcam | ab110413 | 15H4C4 |
| MPO | IHC | Abcam | ab208670 | EPR20257 |
| β-tubulin | WB | BOSTER | BM1453 | TUB2.1 |
| β-actin | WB | Abclonal | AC026 | ARC5115-01 |

**Supplementary Reference**

1. Ding Q, Cao F, Lai S *et al*. Lactobacillus plantarum ZY08 relieves chronic alcohol-induced hepatic steatosis and liver injury in mice via restoring intestinal flora homeostasis. *Food Res Int* 2022;**157**:111259.

2. Donohue TM, Osna NA, Clemens DL. Recombinant Hep G2 cells that express alcohol dehydrogenase and cytochrome P450 2E1 as a model of ethanol-elicited cytotoxicity. *Int J Biochem Cell Biol* 2006;**38**:92−101.

3. Petrosyan A, Cheng PW, Clemens DL *et al*.. Downregulation of the small GTPase SAR1A: a key event underlying alcohol-induced Golgi fragmentation in hepatocytes. *Sci Rep* 2015;**5**:17127.

4. Rabiee A, Plucinska K, Isidor MS *et al*. White adipose remodeling during browning in mice involves YBX1 to drive thermogenic commitment. *Mol Metab* 2021;**44**:101137.

5. Oyama K, Zeeb V, Yamazawa T *et al*. Heat-hypersensitive mutants of ryanodine receptor type 1 revealed by microscopic heating. *Proc Natl Acad Sci U S A* 2022;**119**:e2201286119.
